# G6PC3 promotes genome maintenance and is a candidate mammary tumor suppressor

**DOI:** 10.1172/jci.insight.186747

**Published:** 2025-04-22

**Authors:** Xin Li, Maria Rossing, Ana Moisés da Silva, Muthiah Bose, Thorkell Gudjónsson, Jan Benada, Jayashree Thatte, Jens Vilstrup Johansen, Judit Börcsök, Hanneke van der Gulden, Ji-Ying Song, Renée Menezes, Asma Tajik, Lucía Sena, Zoltan Szallasi, Morten Frödin, Jos Jonkers, Finn Cilius Nielsen, Claus Storgaard Sørensen

**Affiliations:** 1 Biotech Research and Innovation Centre, University of Copenhagen, Copenhagen, Denmark.; 2 Center for Genomic Medicine, Rigshospitalet, and; 3 Department of Clinical Medicine, University of Copenhagen, Copenhagen, Denmark.; 4 Division of Molecular Pathology, The Netherlands Cancer Institute, Amsterdam, Netherlands.; 5 Oncode Institute, Utrecht, Netherlands.; 6 Translational Cancer Genomics Group, Danish Cancer Institute, Copenhagen, Denmark.; 7 Biostatistics Centre & Division of Psychosocial Research and Epidemiology, The Netherlands Cancer Institute, Amsterdam, Netherlands.; 8 Computational Health Informatics Program, Boston Children’s Hospital, Boston, Massachusetts, USA.; 9 Department of Bioinformatics, Semmelweis University, Budapest, Hungary.

**Keywords:** Cell biology, Clinical Research, Genetics, Breast cancer, DNA repair, Genetic instability

## Abstract

Mutations in genome maintenance factors drive sporadic and hereditary breast cancers. Here, we searched for potential drivers based on germline DNA analysis from a cohort consisting of patients with early-onset breast cancer negative for *BRCA1/BRCA2* mutations. This revealed candidate genes that subsequently were subjected to RNA interference–based (RNAi-based) phenotype screens to reveal genome integrity effects. We identified several genes with functional roles in genome maintenance, including Glucose-6-Phosphatase Catalytic Subunit 3 (*G6PC3*), *SMC4*, and *CCDC108*. Notably, G6PC3-deficient cells exhibited increased levels of γH2AX and micronuclei formation, along with defects in homologous recombination (HR) repair. Consistent with these observations, G6PC3 was required for the efficient recruitment of BRCA1 to sites of DNA double-strand breaks (DSBs). RNA-Seq analysis revealed that G6PC3 promotes the expression of multiple homologous recombination repair genes, including *BRCA1*. Through CRISPR-Select functional-genetic phenotype analysis of *G6PC3* germline mutations, we identified 2 germline *G6PC3* variants displaying partial loss of function. Furthermore, our study demonstrated that *G6pc3* deficiency accelerates mammary tumor formation induced by *Trp53* loss in mice. In conclusion, our cohort-based functional analysis has unveiled genome maintenance factors and identified *G6PC3* as a potential candidate tumor suppressor in breast cancer.

## Abstract 2

This study identifies putative tumor suppressors supporting genome maintenance, with a functional focus on G6PC3.

## Introduction

Cellular DNA is constantly challenged by intracellular reactive by-products and environmental stressors, posing a threat to genome stability and promoting tumorigenesis. Among all cancer types, breast cancer holds the highest incidence rate in women (1). Genetic predisposition is a risk factor that accounts for approximately 10% of all patients with breast cancer (2). Patients with hereditary breast cancer (HBC) are frequently diagnosed with mutations in established DNA damage response (DDR) genes, including *BRCA1/2*, *PALB2*, *ATM*, and *CHEK2*, with the majority involved in homologous recombination (HR) repair (3).

Genome maintenance relies on the coordinated function of numerous factors involved in diverse processes. Alongside well-established DDR genes, emerging evidence suggests that a subset of cellular metabolism genes also play a role in regulating genome stability; however, their effect and disease relevance remain poorly explored. For instance, Fumarate hydratase (FH), responsible for converting fumarate to L-malate in the tricarboxylic acid cycle, may regulate HR repair by influencing histone methylation (4). Phosphoglycerate mutase 1 (PGAM1), a glycolytic enzyme involved in various metabolic pathways such as glycolysis, the pentose phosphate pathway (PPP), and serine biosynthesis, may disrupt ATM signaling, consequently impairing HR repair (5). Another glycolytic gene, pyruvate kinase M2 (PKM2), also plays a role in modulating HR repair by regulating CtIP recruitment at DSB sites (6). Similarly, nuclear 6-Phosphofructo-2-Kinase/Fructose-2,6-Biphosphatase 3 (PFKFB3) can enhance HR repair in cancer cells by modulating cellular deoxyribonucleotide triphosphate (dNTP) levels (7). Through independent genetic screens, another glycolytic gene, *G6PC3*, has recently appeared as a candidate factor that may promote HR repair (8, 9); however, its role and relevance in HR repair or genome maintenance remains unknown.

The transmembrane protein glucose-6-phosphatase catalytic subunit 3 (G6PC3) interacts with the glucose-6-phosphate transporter (G6PT) and operates in the final step of gluconeogenesis and glycogenolysis. Here, it hydrolyzes glucose 6-phosphate (G6P) to maintain cellular glucose levels (10, 11). G6PC3 is primarily localized in the endoplasmic reticulum membrane and is expressed in various tissues, including skeletal muscle, heart, blood, and breast. Homozygous germline *G6PC3* loss-of-function variants lead to myeloid cell dysfunction, resulting in severe congenital neutropenia type 4 (SCN4) or Dursun syndrome (12–14). In this disorder, inactivation of G6PC3 leads to the accumulation of noncanonical 1,5-anhydroglucitol-6-phosphate (1,5AG6P) metabolites, which may inhibit glycolysis and consequently reduce the number of neutrophils (15). Furthermore, G6PC3 deficiency has been linked to nonhematologic defects, such as prominent superficial venous patterns, congenital cardiac abnormalities, genitourinary malformations, and thrombocytopenia (16). However, there are no reported associations between *G6PC3* gene mutations and cancer.

In this study, we employed phenotype screening for genome maintenance functions to identify potential breast cancer drivers. We uncovered several factors with putative roles in genome maintenance, and our functional analysis identified a role for G6PC3 in HR repair. Furthermore, loss of *G6pc3* accelerated mammary tumorigenesis in mice, indicating a tumor-suppressor role for G6PC3 in vivo.

## Results

### Functional screen identifies G6PC3 as a potential genome maintenance factor.

Mutated genome maintenance factors are drivers of both familial and sporadic breast cancers. Patients with early-onset breast cancer often present with more aggressive disease and experience worse clinical outcomes (17). Additionally, early-onset breast cancer may indicate the presence of highly pathogenic variants in genes that predispose individuals to the disease. Thus, to identify novel genome maintenance genes and potential breast cancer genes, we analyzed genetic variants in 135 patients with early-onset breast cancer who were *BRCA1/BRCA2* WT (Supplemental Figure 1A; supplemental material available online with this article; https://doi.org/10.1172/jci.insight.186747DS1). We filtered the data to focus on genes enriched for rare variants, requiring at least 1 nonsense/frameshift variant and 1 missense variant per gene. Variants with an allele frequency greater than 1% in public variant databases were excluded, as previously described (18). This filtering process resulted in a shortlist of 150 candidate genes.

**Figure 1:**
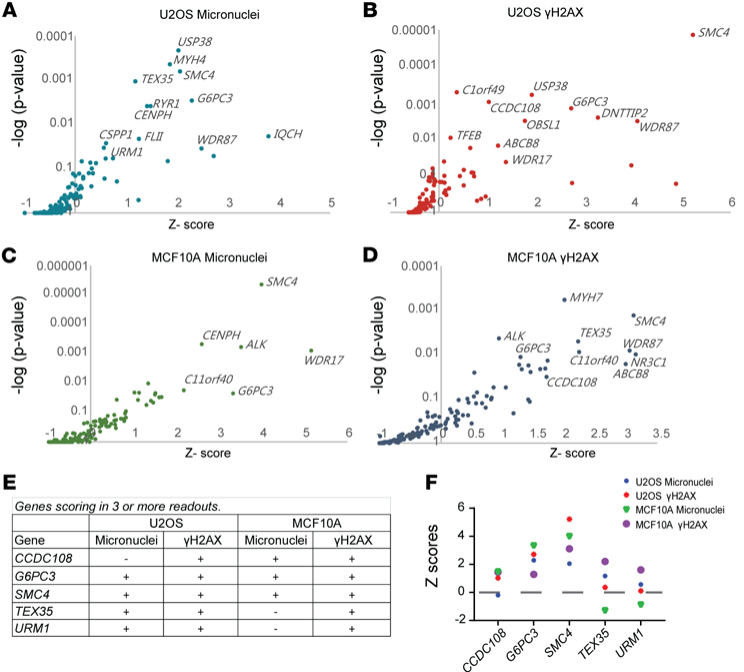
Functional screen identifies G6PC3 as a potential genome maintenance factor. (**A**–**D**) An overview of the siRNA screen output in U2OS (**A** and **B**) and MCF10A (**C** and **D**) cells. The siRNA screens were performed using a siRNA library synthesized from Ambion, with 3 siRNAs targeting each gene (450 in total, 10 nM siRNA). The *y* axis is –log_10_ transformed *P* values, the *x* axis shows z scores based on the average from 3 independent siRNAs. Data analysis was based on percentage of γH2AX^+^ and micronuclei^+^ cells per well. (**E**) Genes that scored significantly in more than 3 siRNA phenotypic readouts. Screen readouts in which the indicated gene score is labeled in “+” as positive, while the “–” symbol indicates no difference compared with negative controls. (**F**) Normalized z score of γH2AX and micronuclei of all the indicated genes in both U2OS and MCF10A cell lines. G6PC3 and SMC4 displayed relatively higher γH2AX and micronuclei z scores in both U2OS and MCF10A cell lines.

We generated a targeted siRNA library for the 150 shortlisted genes, using 3 different siRNAs per gene (Supplemental Figure 1A). This siRNA library was screened for aberrant genome maintenance in 2 human cell lines: nonmalignant breast epithelial cells (MCF10A) and osteosarcoma cells (U2OS). Five days after transfection, we assessed increased γH2AX and micronuclei formation, both of which are markers of genome instability. γH2AX serves as a phosphorylation marker, indicating activated DDR signaling (19, 20) (Supplemental Figure 1B and Supplemental Table 1). The screen identified 5 genes — *G6PC3*, *SMC4*, *CCDC108*, *TEX35*, and *URM1* — that scored in at least 3 of 4 phenotypic readouts (Figure 1, A–E). Among these, SMC4 and G6PC3 knockdown showed a significant increase in γH2AX and micronuclei formation in both cell lines (Figure 1F; additional data in Supplemental Figure 1, C–J).

*SMC4* encodes a protein involved in mitosis (21). Its expression has been linked to breast cancer prognosis and suggested as a prognostic biomarker (22, 23). G6PC3 catalyzes the hydrolysis of glucose-6-phosphate in the final steps of gluconeogenesis and glycogenolysis pathways. However, its role in genome maintenance or cancer is not yet established. Notably, *G6PC3* was a top-ranked hit in 2 independent phenotype screens for HR repair factors (8, 9). Given this, we focused our subsequent efforts on further exploring the functions of G6PC3.

### G6PC3 promotes genome stability.

**Figure 2:**
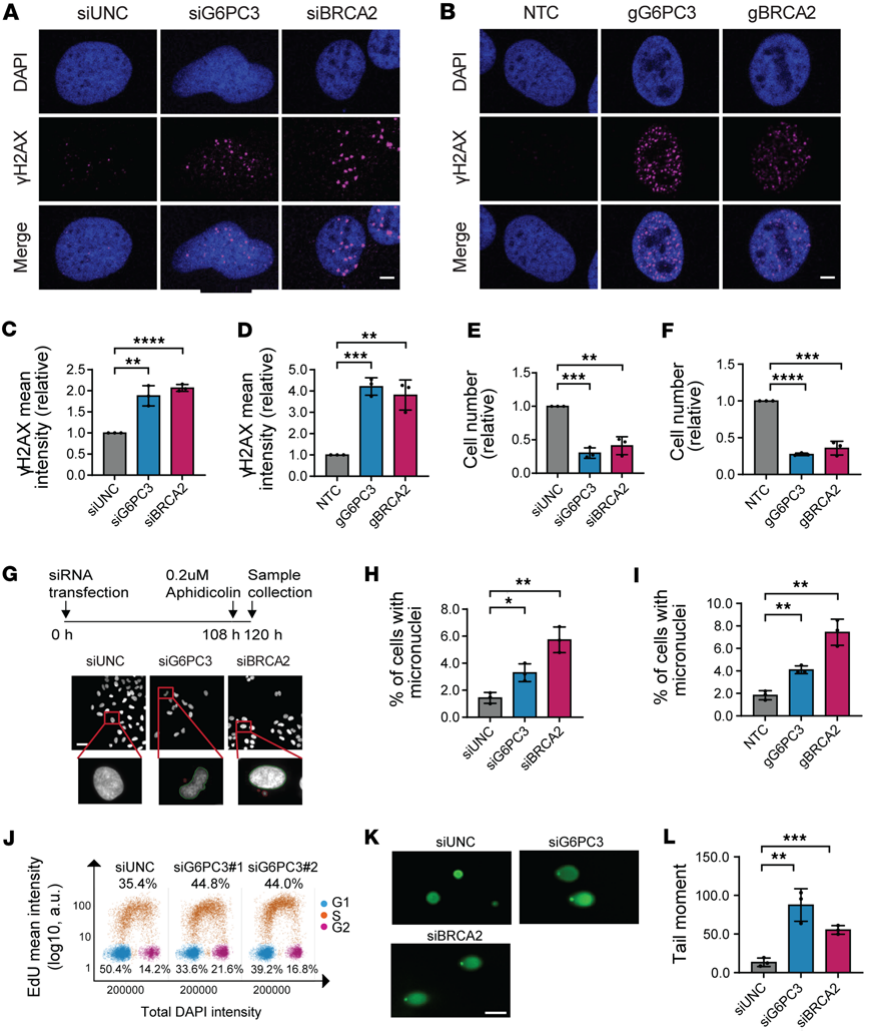
G6PC3 is necessary to maintain genome integrity. (**A** and **B**) Representative confocal images of γH2AX in U2OS cells treated with either siRNAs (**A**) or CRISPR gRNAs (**B**). Scale bar: 5 μm. (**C** and **D**) Bar plots indicate relative γH2AX levels in U2OS cells treated with either siRNAs (**C**) or CRISPR gRNAs (**D**). Fold changes were normalized to control siRNA (set to 1). (**E** and **F**) Bar plots indicate relative cell number in U2OS cells treated with either siRNAs (**E**) or CRISPR gRNAs (**F**). Fold changes were normalized to control siRNA (set to 1). (**G**) Schematic illustration of micronuclei estimation through QIBC. Scale bar: 50 μm. (**H** and **I**) Bar plots indicate percentage of U2OS cells with micronuclei. Sells were treated with siRNAs (**H**) or CRISPR gRNAs (**I**). (**J**) Cell cycle analysis of U2OS cells treated with siRNAs. Cells were gated into G1, S, and G2 phase based on total DAPI and mean EdU intensity. Representation of 1 of 3 biological replicates, *n* = 25,929 per sample. (**K**) Representative images of comet tails in U2OS cells after transfection with indicated siRNAs. Scale bar: 100 μm. (**L**) Bar plot indicate average tail moment, quantified by ImageJ software (67). Data are means ± SD from 3 biological replicates; statistical significance of differences was evaluated using 1-way ANOVA followed by Dunnett’s test. *P < 0.05, **P < 0.01, ***P < 0.001, ****P < 0.0001.

To uncover the roles of G6PC3 in genome maintenance, we analyzed γH2AX signaling in U2OS cells using both siRNA and CRISPR/Cas9 gRNA targeting G6PC3. For comparison, we performed parallel analyses with depletion of BRCA2*,* a key HR gene. Consistent with the phenotypic siRNA screening, downregulation of G6PC3 led to impaired genomic stability, evidenced by increased γH2AX formation (Figure 2, A–D). siRNA knockdown efficiency was estimated by quantitative PCR (qPCR) and shown in Supplemental Figure 2A. Consistently, G6PC3 depletion resulted in elevated γH2AX levels in breast cancer cell lines, including MCF7 and M.D. Anderson - Metastatic Breast 231 (MDA-MB-231) (Supplemental Figure 2, B–E). Besides impaired genomic stability, a decreased cell number was observed upon G6PC3 depletion in all 3 cell lines (U2OS, MCF7, and MDA-MB-231) (Figure 2, E and F, and Supplemental Figure 2, F and G). To further assess cell viability upon G6PC3 depletion, we examined apoptosis using the annexin V kit (24, 25). In comparison with siPLK1 (26), no significant increase in apoptotic cells was observed upon G6PC3 depletion (Supplemental Figure 2, H and I). These results suggest that the cell fitness defects following G6PC3 depletion are not mediated through apoptosis.

Micronuclei are cytoplasmic bodies containing damaged chromosomal fragments or entire chromosomes, commonly used as biomarkers for genomic instability (27). Therefore, we quantified the percentage of micronuclei^+^ cells upon G6PC3 depletion (Figure 2G) using quantitative image-based cytometry (QIBC). Both siRNA- and gRNA-mediated G6PC3 depletion significantly increased micronuclei^+^ cells, comparable with BRCA2 depletion (Figure 2, H and I). Monitoring cell proliferation after EdU treatment revealed that G6PC3 depletion cells did not significantly affect cell cycle progression, except for a slight increase in the proportion of cells in the S phase (Figure 2J). Therefore, we speculate that G6PC3 depletion is more likely to affect cell viability rather than reduce cell cycle progression. Moreover, unresolved DNA breaks, known to induce genomic instability, were assessed using the Comet assay (28) upon G6PC3 depletion, which significantly increased the tail moment (Figure 2, K and L). Notably, in all these analyses, depletion of BRCA2 produced similar effects. These findings collectively indicate the involvement of G6PC3 in genome maintenance.

To further investigate G6PC3 functions, we generated cell lines with inducible GFP-G6PC3 and FLAG-G6PC3 expression. The protein expression was validated by immunoblotting, with G6PC3 marked by the red triangle (Supplemental Figure 3A). As previous studies have shown (10), G6PC3 predominantly localizes to the cytoplasmic compartment rather than the nucleus (Supplemental Figure 3B). Surprisingly, we observed that overexpression of G6PC3 in our inducible cell lines led to increased γH2AX formation (Supplemental Figure 3, B–D). This phenomenon was not observed in cells transfected with an empty GFP vector or control cells (Supplemental Figure 3, B–D). These findings imply that maintaining a balanced expression of G6PC3 promotes genome integrity.

### G6PC3 promotes HR repair.

**Figure 3:**
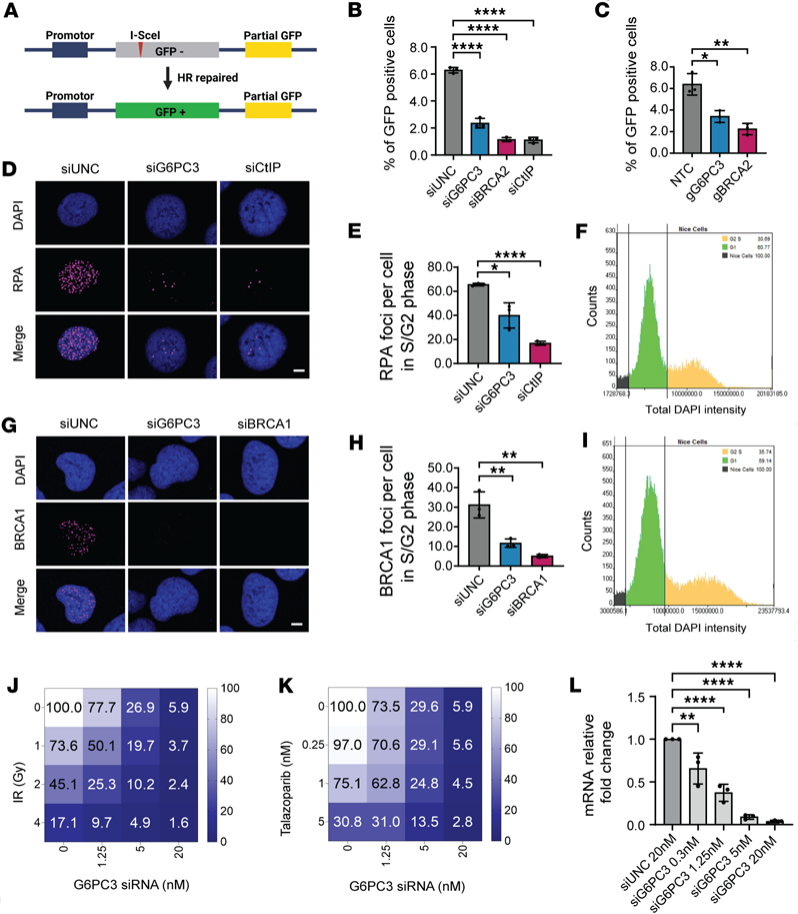
G6PC3 promotes homologous recombination. (**A**) Schematic illustration of the DR-GFP assay to measure homologous recombination efficiency. (**B** and **C**) Flow cytometry–based quantification of HR efficiency in U2OS cells after siRNA (**B**) or CRISPR gRNAs treatment (**C**). Ratios were normalized to the values obtained with control siRNA (siUNC) or nontreated cells (NTC). (**D**) Representative confocal images of RPA in U2OS cells. Scale bar: 5 μm. Cells were treated with indicated siRNA (30 nM) for 48 hours. DNA DSBs were induced using 5 Gy IR, and cells were fixed and imaged after 6 hours. (**E**) Bar plot indicate RPA foci levels (per cell) in S/G2 phase. (**F**) Representative image of cell cycle estimation by QIBC for RPA foci analysis. (**G**) Representative confocal images of BRCA1 in U2OS cells. Experiments were performed as in **D**, except DNA DSBs were induced using 2 Gy IR and cells were fixed and imaged after 1 hour. (**H**) Bar plot indicate BRCA1 foci levels (per cell) in S/G2 phase. (**I**) Representative image of cell cycle estimation by QIBC for BRCA1 foci analysis. (**J** and **K**) Dose response matrix for cell viability 5-day treatment after IR (**J**) or Talazoparib (**K**) in combination with G6PC3 siRNA in U2OS cells; data represent mean from triplicate. (**L**) qPCR was used to determine G6PC3 expression. U2OS cells were transfected with different concentration of siRNA for 48 hours. Data are shown as mean ± SD from 3 biological replicates; statistical significance of differences was evaluated using 1-way ANOVA followed by Dunnett’s test. *P < 0.05, **P < 0.01, ****P < 0.0001.

The high-fidelity HR repair pathway suppresses genome instability; it particularly deals with DNA double-strand breaks (DSBs) occurring during the S and G2 phases of the cell cycle (29). To investigate whether G6PC3 is involved in HR repair (HRR), we utilized a GFP fluorescence-based cellular HRR reporter assay (Figure 3A) (30). Similar to the depletion of known HRR genes, *BRCA2* and *CtIP*, the depletion of G6PC3 significantly decreased the efficiency of HRR in cells (Figure 3, B and C, and Supplemental Figure 3E).

To gain a deeper understanding of the underlying mechanism, we evaluated the function of several key HRR genes after depleting G6PC3 in cells. When cells utilize the high-fidelity HRR pathway to repair harmful DSBs, the MRN complex (comprising MRE11, RAD50, and NBS1) initiates DNA end resection, with RPA subsequently playing a critical role by binding to the 3′ single-stranded DNA (ssDNA) on the resected DNA DSBs (31). As demonstrated in Figure 3, D and E, ionizing radiation–induced (IR-induced) damage resulted in substantial RPA accumulation at DSBs in negative control (siUNC) cells, whereas RPA foci were markedly reduced in G6PC3-knockdown cells. BRCA1, another crucial HRR factor, collaborates with carboxy-terminal binding protein in *RBBP8* (CtIP) in DNA end resection and promotes RAD51 recruitment during the late stage of HRR (32, 33). Our study revealed that, following IR damage, *G6PC3* knockdown led to a significant reduction in *BRCA1* foci formation at DSBs (Figure 3, G and H). Importantly, we monitored RPA and BRCA1 foci formation in cells during the S and G2 phases, ensuring that these differences were not attributable to variations in the cell cycle (Figure 3, F and I). In summary, our results imply that G6PC3 plays a role in HRR.

DNA-damaging agents are used to target tumors with HR defects. Given that G6PC3 knockdown led to decreased HR repair efficiency, we aimed to determine whether depleted cells exhibited increased sensitivity to DNA repair–related drugs or treatments. To explore this, we transfected cells with different concentrations of G6PC3 siRNA and subsequently treated them with the PARP inhibitor Talazoparib or IR to assess cellular sensitivity. As shown in Figure 3, J and K, G6PC3 depletion sensitized cells to both IR and Talazoparib, with a more pronounced effect at higher siRNA concentrations. The knockdown efficiency of different siRNA concentrations is presented in Figure 3L. Altogether, these findings further suggest that G6PC3 promotes HRR.

### G6PC3 promotes transcription of HRR pathway genes.

**Figure 4:**
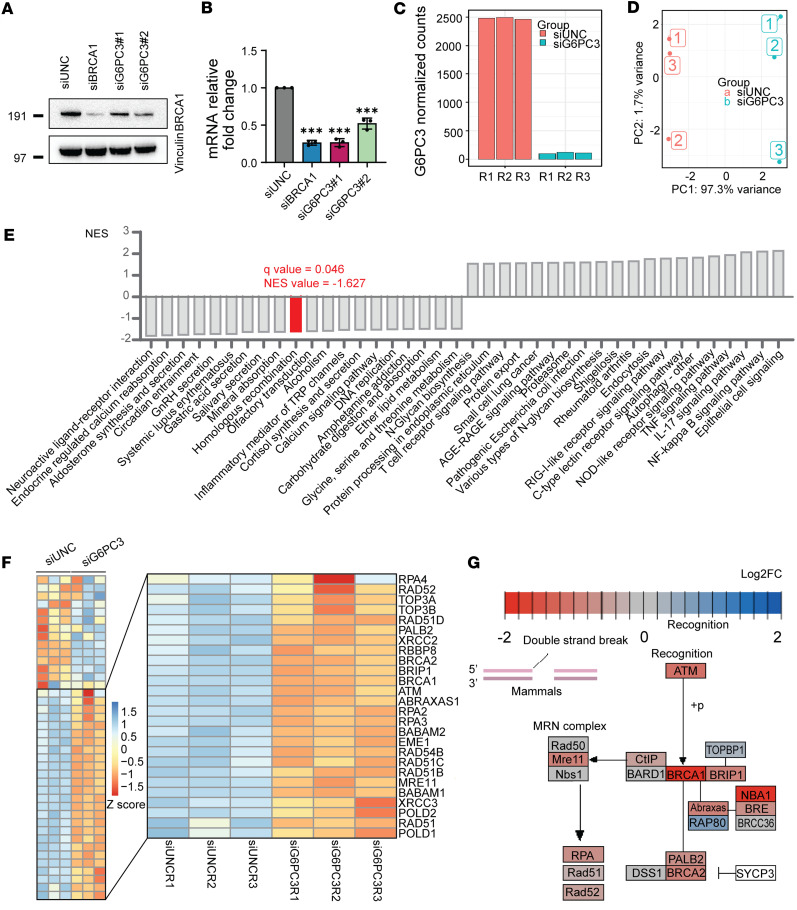
G6PC3 promotes transcription of HRR genes. (**A**) Representative Western blots for BRCA1 expression. U2OS cells were transfected by different siRNA, and the lysate was collected 48 hours afterward. Endogenous actin was used as a loading control. (**B**) qPCR was used to determine BRCA1 aberrant expression. U2OS cells were transfected with different siRNA for 48 hours. Bar plot indicates means ± SD from 3 biological replicates; statistical significance of differences was evaluated using 1-way ANOVA followed by Dunnett’s test. ***P < 0.001. (**C**) Validation of effective G6PC3 siRNA knockdown from RNA-Seq datasets. Bar plot representing the DESeq2-normalized G6PC3 counts in each U2OS sample used for RNA-Seq analysis. (**D**) PCA plot for top 500 variable genes across all samples. (**E**) GSEA of top 20 upregulated and downregulated pathways after G6PC3 siRNA depletion (*q* < 0.1). The *y* axis represents KEGG pathways; the *x* axis represents the normalized enrichment score (NES) for each pathway. (**F**) Heatmap illustrates differential expressed HR genes between G6PC3 and control siRNA-treated U2OS cells. Twenty-six downregulated HR genes after G6PC3 depletion are annotated in the figure. The heatmap is colored according to the z score of each gene normalized by row. (**G**) Analysis of HR pathway (KEGG). The genes are colored according to their log_2_FC between G6PC3 and control siRNA treated U2OS from the DESeq2 statistical analysis. PCA, Principal component analysis; GSEA, gene set enrichment analysis.

G6PC3 may influence the recruitment of BRCA1 to chromatin in various ways, such as by promoting the levels of BRCA1 mRNA and protein. To investigate this notion, we examined BRCA1 protein and mRNA expression following G6PC3 depletion using immunoblotting and qPCR. As shown in Figure 4, A and B, loss of G6PC3 resulted in a significant downregulation of BRCA1 protein and mRNA levels. To further validate these findings, we conducted transcriptome analysis in G6PC3-depleted cells using RNA-Seq (Figure 4, C and D). Gene set enrichment analysis (GSEA) based on RNA-Seq data demonstrated a significant downregulation of the HRR pathway following G6PC3 depletion (Figure 4E). Additionally, multiple HRR genes showed suppression in the absence of G6PC3 (Figure 4F), with log_2_ fold change (log_2_FC) values indicating *BRCA1* as the most downregulated gene (Figure 4G). These results strongly suggest that G6PC3 mediates the accumulation of BRCA1 and other HRR proteins.

Furthermore, we explored if the G6PC3 effect on BRCA1 function and expression (Figure 3G and Figure 4, A and B) was unique or if it was linked to impairment in the glycogenolysis/gluconeogenesis pathway. Therefore, we examined BRCA1 foci and protein levels following the depletion of various glycogenolysis/gluconeogenesis genes, including *HK2*, *G6PT*, and *PFKP*. Unlike G6PC3, depletion of HK2 and PFKP did not significantly reduce BRCA1 foci levels (Supplemental Figure 5A). Additionally, depletion of G6PT, HK2, and PFKP did not affect BRCA1 protein levels (Supplemental Figure 5B), which differed from the decrease observed in both G6PC3 and BRCA1 siRNA–treated cells (Figure 4, A and B, and Supplemental Figure 5, B and C). Interestingly, depletion of G6PT, an interacting partner of G6PC3, displayed a modest decrease in BRCA1 foci levels (Supplemental Figure 5A). Together, these findings could indicate that the G6PC3 role in genome maintenance and DNA repair may differ from its canonical catalytical function in G6P hydrolysis.

### Functional validation uncovers 2 potentially deleterious G6PC3 variants.

Initially, we identified 2 germline *G6PC3* variants in the early-onset breast cancer cohort, as shown in Supplemental Figure 4A. Subsequently, we screened for *G6PC3* variants in a larger cohort of unselected patients with breast cancer and identified an additional 7 variants (Supplemental Figure 4A). To investigate the effect of these *G6PC3* variants, we utilized the quantitative CRISPR-Select assay to evaluate the functional genetic phenotypes of these variants (Supplemental Figure 4B) (34). Three of the 9 variants have been previously reported in patients with SCN4 (ClinVar), so we did not investigate these further. Given that G6PC3 depletion affected cell viability and that HR is essential (Figure 2, E and F, and Supplemental Figure 2, E and F), we employed the CRISPR-Select-TIME assay to track frequencies of the *G6PC3* variants in U2OS cells over 12 days based on NGS. We transfected cells with a CRISPR-Select cassette containing target-specific CRISPR-Cas9 components and 2 single-stranded oligodeoxynucleotide (ssODN) repair templates to create *G6PC3* variants in the same dish. The *G6PC3* ssODN templates differed only in that one harbors the variant of interest while the other featured a synonymous internal mutation (WT’) for normalization. We then compared the variant/synonymous mutation (WT’) ratios early with later time point, which allowed us to determine if *G6PC3* variants were selectively depleted as observed for HR-deficient *BRCA2* variants (29). As shown in Supplemental Figure 4B, the p.F43V and p.P169S variants significantly reduced cell fitness, indicating that these 2 mutations impaired G6PC3 function. Since *G6PC3* encodes a ubiquitously expressed enzyme that hydrolyzes G6P into glucose and phosphate during glycogenolysis and gluconeogenesis, we questioned whether the effect of G6PC3 on cell fitness was due to its enzymatic activity. To test this, we again utilized the CRISPR-Select-TIME assay to evaluate 3 catalytically dead *G6PC3* variants (p. R79A, p. H114A, and p. H167A) (10). As shown in Supplemental Figure 4C, none of these enzymatic mutations affected cell viability, suggesting that the phenotypes observed in Supplemental Figure 4B were not caused by the loss of G6PC3 enzymatic activity. Next, we explored if G6PC3 may contribute to somatic breast cancer–related clinical outcomes. First, we examined whether G6PC3 was mutated in the publicly available sporadic cancer dataset, The Cancer Genome Atlas (TCGA) breast cancer (BRCA) cohort, but no mutations were identified. We then investigated whether G6PC3 expression correlated with overall survival by analyzing its expression across different breast cancer hormone receptor subtypes with focus on BRCA1/2-intact patients. Although low G6PC3 expression appeared to correlate with improved overall survival probability, this association was not statistically significant, possibly due to the relatively small number of samples with reduced G6PC3 expression (Supplemental Figure 4, D–F).

### G6PC3 suppresses mammary epithelium tumorigenesis following TP53 loss.

**Figure 5:**
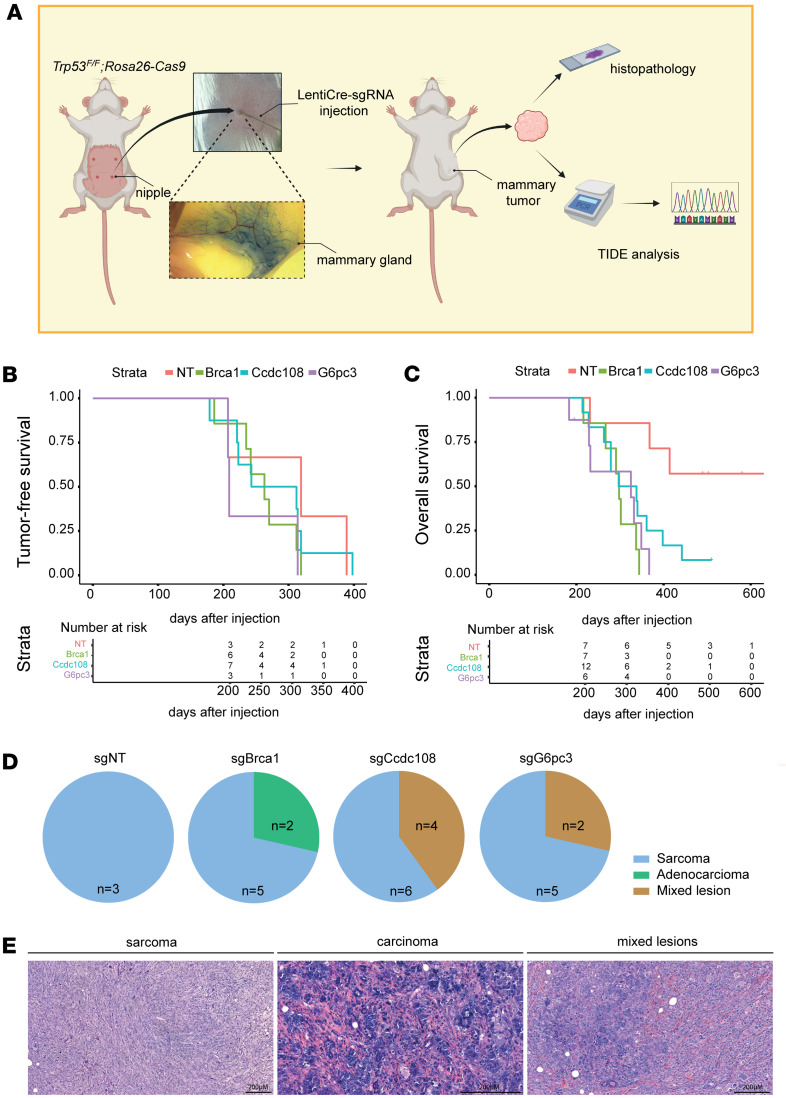
KO of potential genome maintenance factors accelerates mammary tumorigenesis in a *Trp53*-deficient somatic breast cancer mouse model. (**A**) Schematic diagram of somatic mouse models of breast cancer, intraductally injected with high-titer lentiviruses encoding Cre and nontargeting (NT) sgRNA, or sgRNAs targeting *Brca1*, *Ccdc108*, or *G6pc3* alleles in *Rosa26-Cas9;Trp53^fl/fl^* females. *G6pc3* KO results in tumor formation. Tumors were harvested and undergo histopathology and TIDE analysis. (**B**) Kaplan-Meier analysis of mammary tumor-free survival of *Rosa26-Cas9;Trp53^fl/fl^* mice injected with sgNT (sgBrca1 vs sgNT: *P* = 0.271; sgCcdc108 vs sgNT: *P* = 0.620; sgG6pc3 vs sgNT: *P* = 0.190, Cox proportional hazards model). The different groups don’t show a significant difference in tumor onset. The number of animals at risk over time is represented in the bottom table. One animal in the *Brca1* cohort and 1 in the *Ccdc108* aren’t represented because they were euthanized due a tumor before 200 days after injection. (**C**) Kaplan-Meier analysis of overall survival of *Rosa26-Cas9;Trp53^fl/fl^* females injected with sgNT (*n* = 7), sg*Brca1* (*n* = 7), sg*Ccdc108* (*n* = 12), and sg*G6pc3* (*n* = 8). (sgBrca1 vs sgNT: *P* = 0.00250; sgCcdc108 vs sgNT: *P* = 0.02044; sgG6pc3 vs sgNT: *P* = 0.00431, Cox proportional hazards model). The number of animals at risk over time is represented in the bottom table. Two mice in the *G6pc3* cohort aren’t represented because they were euthanized due a tumor before 200 days after injection. (**D**) Histopathological classification of the main tumor of each mouse injected with sgNT (*n* = 3), sg*Brca1* (*n* = 7), sg*Ccdc108* (*n* = 10), or sg*G6pc3* (*n* = 7). (**E**) H&E stained representative images of the 3 different tumor types present in the cohorts, sarcoma (sgG6pc3 tumor no. 2211174), carcinoma (sgBrca1 tumor no. 2218975), and mixed lesion (sgG6pc3 tumor no. 2312753). Scale bar, 200 μm.

Based on our observation that G6PC3 plays a role in genome maintenance and that mutations may contribute to familial breast cancer, we further investigated the potential tumor-suppressive function of G6PC3 in vivo in mammary epithelial cells. To this end, we generated a somatic mouse model, as described before (35). To induce simultaneous mammary gland–specific loss of *Trp53* and the target gene, we intraductally injected lentiviral vectors (LV) encoding cre recombinase and a single-guide RNA (sgRNA) (LV-Cre+sgRNA) into the mammary gland of female genetically engineered mice expressing Cas9 (*Trp53^fl/fl^;R26-Cas9*) (Figure 5A). Somatic inactivation of *Trp53* contributes to mammary tumor formation in breast cancer mouse models (36). In addition to LV-Cre+sg*Gcpc3*, we produced sgRNAs targeting *Ccdc108*, another hit from our initial screen (Figure 1, E and F), and *Brca1*, a well-known susceptibility gene used as a positive control, along with a nontargeting (NT) sgRNA. Groups of 7–12 mice were injected with the different sgRNAs, and we monitored their tumor latency over the ensuing period. Mice injected with LV-Cre+sgNT developed tumors induced by the loss of *Trp53* alone, with a median tumor-free survival of 319 days. In contrast, animals injected with sg*Brca1*, sg*Ccdc108*, or sg*G6pc3* presented tumors at 263 days, 277.5 days, and 209 days, respectively. Tumors detected for the first time at a large size (>62.5mm^3^) were excluded from this analysis. When compared with the NT group, none of the other groups showed a significant difference in tumor-free survival (*Brca1*: *P* = 0.271; *Ccdc108*: *P* = 0.620; *G6pc3*: *P* = 0.190, Cox proportional hazards model) (Figure 5B). LV-Cre+sg*Brca1*–injected females, with a median overall survival of 298 days, exhibited a significantly acceleration in mammary tumor formation compared with the loss alone of *Trp53* (490 days; *P* = 0.00250). Intriguingly, intraductal injections of LV-Cre+sg*G6pc3* and +sg*Ccdc108* resulted in shorter median overall survival, 278.5 days (*P* = 0.00431) and 317.5 days (*P* = 0.02044), respectively, compared with the NT group (Figure 5C). Of the 7 mice injected with LV-Cre+sgNT, only 3 developed tumors and were included in the overall survival analysis. Four animals were either found dead in a cage or had to be euthanized due to other health issues, while 1 mouse remained alive at the end of the in vivo study. In each of the *G6pc3* and *Ccdc108* groups, 1 animal was found dead in its cage. Mice were euthanized when tumor volume reached 1,500 mm^3^. To assess disruption of the targeted genes, Tracking of Indels by Decomposition (TIDE) analysis was conducted on samples from the largest tumor of each mouse (main tumor) (Supplemental Figure 6A and Supplemental Table 8), alongside PCR analysis (Supplemental Figure 6B). Histological analysis of all main tumors revealed that tumors induced by *Trp53* loss alone were exclusively sarcomas, while the group with the additional loss of *Brca1* was the only one developing pure adenocarcinomas. Most of the tumors resulting from dual loss of *G6pc3* or *Ccdc108* and *Trp53* were sarcomas; however, mixed lesions comprising both sarcomas and carcinomas were also present in these groups (Figure 5, D and E, and Supplemental Table 9). We focused our analysis on fully advanced tumors, excluding initial lesions from consideration.

## Discussion

Among the risk factors contributing to breast cancer, genetic predisposition accounts for approximately 10% (37), and HBC is often associated with mutations in genome maintenance genes (38). Through prior high-throughput sequencing data (18), we filtered and selected genes for phenotypic siRNA screening to identify potentially novel HR regulators and potential HBC genes. Our phenotypic screening approach identified potential genome maintenance roles for CCDC108, G6PC3, SMC4, and TEX35 depletion (Figure 1, E and F). G6PC3-depleted cells exhibited a significant decrease in HRR efficiency, a key pathway in breast cancer tumor suppression. Intriguingly, G6PC3 promoted RPA and BRCA1 accumulation at DNA damage sites. Transcriptomic analysis revealed downregulation of multiple HR factor transcripts in G6PC3-depleted cells. Notably, BRCA1 expression levels were markedly impaired in G6PC3-depleted cells, likely contributing to the observed DNA repair defects. We noted a transcriptional effect on multiple important biological pathways, some of which may contribute to HRR and genome maintenance. For example, the significant downregulation of DNA replication transcripts and upregulation of immune response pathways after G6PC3 depletion could also contribute to genome instability and cell proliferation effects (Figure 4E).

G6PC3 may have additional cancer-relevant roles, as it plays important roles in gluconeogenesis and glycogenolysis pathways. Emerging evidence suggests that some by-products of metabolic reactions can inflict detrimental DNA damage. Shortages in dNTP pools can lead to replication stress, resulting in increased mutagenesis, genomic instability, and eventually tumorigenesis (39, 40). Similar to G6PC3, other glycolytic enzymes such as G6PT, Hexokinase 2 (HK2), and PFKP also play important roles during glycolysis. G6PT regulates the transport of glucose-6-phosphate from the cytoplasm to the lumen of ER to maintain glucose homeostasis. It collaborates with G6PC3 to hydrolyze the glucose-6-phosphate into glucose and phosphate (41). HK2 phosphorylates glucose to produce G6P, which serves as a substrate for G6PC3’s catalytic function. Our results suggest that the loss of subset of glycolytic genes (HK2 and PFKP) may not impair BRCA1 functions (Supplemental Figure 5, B and C); hence, aberrations in the glycolytic pathway alone may not fully account for impaired HRR in G6PC3-depleted cells. Nevertheless, it is well possible that G6PC3 deficiency may affect cell viability through insufficient supply of metabolic components or energy. Aberrant enzymatic activity of G6PC3 leads to excessive accumulation of a noncanonical metabolite, 1,5AG6P, inhibiting glycolysis and resulting in dysfunction of neutrophil cells (15). To gain more insight into how potential deleterious variants affect cell viability, we investigated the connection between G6PC3 metabolic enzyme activity and cell viability. Residues R79, H114, and H167 in G6PC3 are crucial for its catalytic function in glycolysis and neutrophil regulation. Notably, catalytic dead *G6PC3* variants (p. R79A, p. H114A, and p. H167A) did not display changes in cell fitness in our functional assay. Therefore, we consider it likely that impaired HRR, which is essential, contributes to cell fitness issues when G6PC3 is disabled. Moreover, our data indicate that the enzymatic activity of G6PC3 in G6P hydrolysis may be dispensable for its involvement in HRR. Altogether, we infer that *G6PC3* variants may lead to tumorigenesis-promoting impaired HRR function, an effect that is known to markedly affect cell fitness in tissue culture systems.

Patients with germline mutations in high-risk breast cancer genes such as *BRCA1* are predisposed to developing breast cancers with characteristic features, including frequent *TP53* mutations. The somatic conditional mouse model in our study carries tissue-specific KO of *G6pc3* and/or *Trp53* in basal epithelial cells, enabling the investigation of the role of these genes in breast cancer development. Our results demonstrate that somatic loss of both *G6pc3* and *Trp53* accelerated the formation of tumors in mice and reduced the survival rate of mice. Our cancer genomic analysis based on the TCGA database indicate that G6PC3 mutations may be more relevant in familial rather than sporadic instances. In summary, we identified a role of G6PC3 in genome maintenance and HR repair through functional screening and subsequent molecular assays. These findings provide insights into potential tumor suppressors and harmful variants. Similar to *FH*, *PGAM1*, *PKM2*, and *PFKFB3*, *G6PC3* may be considered a noncanonical gene promoting HRR and genome stability.

## Methods

### Sex as a biological variable.

In our mouse models, sex was not considered a biological variable because only female mice were used for cancer induction experiments.

### Breast cancer cohorts and genetic screening.

Detailed information about the early-onset breast cancer cohort has been published in a previous study (18). The unselected breast cancer cohort consists of 1,045 patients with *BRCA* WT breast cancer previously screened through the clinical work-up. Whole exome sequencing (WES), panel-sequencing (including the *G6PC3* gene), and data processing were performed as previously described (42). Rare *G6PC3* variants were filtered and manually curated using the Integrative Genomics Viewer (IGV).

### Arrayed siRNA screen.

The siRNA library targeting 150-selected genes (Supplemental Table 1) was purchased from Ambion (Silencer Select siRNA). Each gene was targeted by 3 independent siRNAs and their location in the 384-well plate was randomized using an ECHO liquid dispenser to avoid positional bias. The arrayed siRNA screen was performed in an automated fashion using the Hamilton STARlet liquid dispenser. On day 0, reverse transfection was performed on MCF10A and U2OS cells using 10 nM silencer select siRNA. Five days after transfection, cells were fixed in 4% formaldehyde (VWR) for 15 minutes. After fixation, cells were washed with PBS 4 times and permeabilized using 0.25 % Triton‑X 100 for 10 minutes. After permeabilization, cells were washed with PBS for 4 times, before blocking in 3% BSA for 1 hour at room temperature. Primary antibody incubation targeting γH2AX (MilliporeSigma, 05‑636, 1:1,000) was performed overnight at 4°C. On the following day, after 4 times of PBS washes, secondary antibody incubation with the anti-mouse Alexa Fluor 488 antibody (Invitrogen, A-21202) was performed for 1 hour at room temperature. Cells were washed with PBS for 4 times and subsequently incubated with 1 μg/mL DAPI for 30 minutes at room temperature. Finally, after DAPI incubation, cells were washed with PBS for 5 times and imaged. Sixteen imaging fields were acquired with an IN Cell Analyzer 2200 microscope and a 20× objective (GE Healthcare) to obtain ~2,000 cells/well for control samples for the analysis. All the subsequent analyses were performed with the IN Cell Analyzer Workstation (Cytiva) software. DAPI staining was used to identify the nuclei and micronuclei using the top-hat segmentation method, and the γH2AX mean intensity was measured within the nuclei. Data analysis was performed as mentioned in ref. 43, based on the percentage of γH2AX and micronuclei positive cells per well. In addition to this, Strictly Standardized Mean Difference (SSMD) was also calculated per gene, based on the normalized levels of γH2AX and micronuclei positive cells (43). The z score was calculated using the following equation Z = (X−μ)/σ, where X is the percentage of γH2AX and micronuclei positive cells from the screens (raw score), μ is the mean of the population, and σ is the standard deviation of the population.

### iCas9 U2OS cells.

To generate inducible Cas9–expressing (iCas9-expressing) U2OS cells, early-passage U2OS cells were transduced with lentivirus-containing doxycycline-inducible Cas9 and blasticidin resistance gene (Horizon, CAS11229). In U2OS cells, lentivirus was added at a multiplicity of infection (MOI) of 2 in the presence of 8 μg/mL polybrene for 24 hours. After 24 hours, media containing lentiviral particles were replaced with fresh media and incubated for an additional 24 hours. After incubation, iCas9 U2OS cells were maintained in growth media supplemented with 5 μg/mL blasticidin for selection.

### Cell culture.

The human osteosarcoma cell line (U2OS), human breast cancer cell line MDA-MB-231, and human embryonic kidney 293 cell line (HEK293T) were cultured in DMEM supplemented with 10% FBS (HyClone, HYCLSV30160.03) and 1% penicillin/streptomycin (Thermo Fisher Scientific, 15140-130). Human nonmalignant breast epithelial cell line MCF-10A was cultured in DMEM F-12 (Thermo Fisher Scientific, 31330095) supplemented with 5% horse serum (Thermo Fisher Scientific, 26050088), 1% penicillin/streptomycin (Thermo Fisher Scientific, 15140-130), 10 μg/mL insulin (Sigma-Aldrich, I1882-100MG), 0.5 μg/mL hydrocortisone (Sigma-Aldrich, H0888-1G), 20 ng/mL EGF (PeproTech, AF-100-15), and 100 ng/mL cholera toxin (MilliporeSigma, C8052-5MG). Human breast cancer cell line Michigan Cancer Foundation-7 (MCF7) was cultured in RPMI 1640 Medium, GlutaMAX Supplement (Thermo Fisher Scientific, 61870036) supplemented with 10% FBS (HyClone, HYCLSV30160.03) and 1% penicillin/streptomycin (Thermo Fisher Scientific, 15140-130).

### siRNA and CRISPR gRNA transfection.

siRNAs were synthesized from Sigma-Aldrich and reconstituted in Tris-EDTA buffer solution (Sigma-Aldrich, 93283-500ML) at 20 μM. For siRNA transfection, 0.5 million cells were seeded on a 6 cm dish. The following day, 30 nM siRNA were transfected using Lipofectamine RNAiMAX (Thermo Fisher Scientific, 13778500) according to the manufacturer’s protocol. The media were changed both before transfection and 5 hours after transfection. siRNA sequences used in this study are provided in Supplemental Table 2. G6PC3 siRNA depletion was performed using siG6CP3#1 unless otherwise specified.

CRISPR gRNAs were purchased in the form of crRNA:tracrRNA duplexes from Integrated DNA Technologies and reconstituted in nuclease-free duplex buffer at 10 μM. For ribonucleoprotein (RNP) generation, Alt-R SpCas9 Nuclease V3 (Integrated DNA Technologies, 1081059) was used. For CRISPR gRNA transfection, 0.5 million iCas9 U2OS cells were seeded on a 6 cm dish, and Cas9 expression was induced using 1 μg/mL doxycycline (MilliporeSigma, D3447). The following day, 30 nM gRNA (30 nM crRNA: 30 nM tracrRNA) or the RNP complex were transfected using Lipofectamine RNAiMAX (Thermo Fisher Scientific, 13778500) according to the manufacturer’s protocol. The media were changed both before transfection and 5 hours after transfection with 1 μg/mL doxycycline containing media. CRISPR RNA (crRNA) sequences used in this study are provided in Supplemental Table 3.

### Immunostaining.

Cells were grown on coverslips (Hampton Research, HR3-231) and treated with siRNAs or CRISPR gRNAs as described above. Cells were fixed in 4% paraformaldehyde for 15 minutes and permeabilized using 0.2% Triton X100 for 5 minutes. For RAD51 and RPA staining, the cells were treated with preextraction buffer (0.5% Triton X-100, 20 mM HEPES [pH 7.4], 100 mM NaCl, 3 mM MgCl_2_, and 300 mM sucrose) for 5 minutes before fixation. Cells were then incubated in blocking buffer (3% BSA, 10 % FBS in DMEM media) for 1 hour at room temperature. The primary antibody was diluted in blocking buffer, and the cells were incubated with the primary antibody for 1 hour at room temperature. Following incubation, the cells were washed 3 times with PBS buffer. The secondary antibody was diluted in blocking buffer, and the cells were incubated with the secondary antibody for 30 minutes at room temperature. Following incubation, the cells were washed 3 times with PBS buffer and stained with DAPI (1 μg/mL) for 15 minutes. After washing, the coverslips were mounted using DAKO fluorescence mounting medium (Agilent, S3023). The primary antibodies used in this study are γH2AX (1:1,000, MilliporeSigma, JBW301), RPA (1:1,000, MilliporeSigma, MABE285), and BRCA1 (1:200, Santa Cruz Biotechnology Inc., sc6954). Alexa Fluor goat anti–mouse 647 (1:1,000, A21241, Invitrogen) was used as the secondary antibody.

### QIBC analysis.

Fluorophore-stained cells were imaged using the motorized Olympus IX-83 wide-field microscope controlled by ScanR software. All the images were acquired using either Olympus Universal Plan Super Apo 40× objective or 20× objective. Preliminary image processing and quantification were done using ScanR image analysis software. Total nuclear pixel intensities for DAPI and mean nuclear intensities for γH2AX was recorded. Foci number surrounding the nucleus for micronuclei analysis and foci number of BRCA1, RAD51, and RPA in the nucleus was estimated. At least 100 images (>500 cells) were recorded per coverslip, and TIBCO Spotfire software was used for subsequent data processing and analysis.

### Alkaline comet assay.

After siRNA treatment for 48 hours, U2OS cells were collected at 1 × 10^5^ cells/mL in ice-cold PBS. Alkaline comet assay was performed using the COMET assay kit (Trevigen, 4250-050-K). Low melting (LM) agarose embedded cells were supported by Gel Bond Film (Lonza, 53734), followed by electrophoresis at 30 V on ice for 30 minutes. The samples were then subjected to SYBR Gold dye, and pictures were taken using Zeiss Axio Imager M2 microscope with a 10× objective. Comet tail moment was analyzed using OpenComet plugin from ImageJ (NIH), and for each sample, at least 50 comets were calculated.

### Apoptosis assay.

To measure the percentage of apoptotic cells 72 hours after siRNA treatment, annexin V–conjugated Alexa Fluor 647 (Invitrogen, A23204), the apoptosis detection kit was used according to the manufacturer’s instructions. Propidium iodide (PI) was used to stain the DNA, and live cells were analyzed using BD Fortesa (BD Biosciences). In total, 10,000 events were recorded for each sample, and apoptotic cells were estimated as shown in Supplemental Figure 2E using FlowJo software.

### HR assay.

U2OS cells, containing a stably integrated pDR-GFP plasmid (Addgene, 26475) (44) was a gift from Jiri Lukas lab, University of Copenhagen, Denmark. The DR-GFP reporter plasmid, originally generated in Maria Jasin’s lab, contains an inactive GFP with an in-frame stop codon and an I-Sce1 restriction site, followed by a partial GFP segment corresponding to the stop codon site. When cells are transfected with the plasmid expressing I-Sce1, I-Sce1 makes a DNA DSB, which is repaired by HR using the partial GFP segment as template, resulting in the activation of GFP. To measure HR efficiency, cells were seeded on a 6 cm dish and, the next day, transfected with siRNA or CRISPR RNP complex. Twenty-four hours after transfection, cells were transfected with I-Sce1 plasmid using GenJet according to the manufacturer’s protocol. Two days after plasmid transfection, cells were fixed with 1% formaldehyde for 15 minutes at room temperature and permeabilized in 60% ethanol for 1 hour on ice. The DNA was stained with PI at 2.5 μg/mL containing RNase A at 25 μg/mL for 30 minutes at 37°C and samples were then analyzed by BD Fortesa (BD Biosciences) and FlowJo software. In total, 10,000 cells were collected for each sample, and HR efficiencies were calculated based on the percentage of GFP^+^ cells, and finally, relative HR efficiencies were obtained by calculating the relative changes in GFP frequency compared with control siRNA cells (control = 1).

### Talazoparib/irradiation sensitivity assay.

Twenty-four hours after siRNA transfection, 800 cells were plated into 96-well plates (Greiner-BIO) at 100 μL volume and treated with talazoparib or irradiated at indicated doses. After 5 days of incubation at 37°C, 30 μL of phosphate-buffered saline (PBS) containing 4 μg/mL Hoechst 33342 and 1/10, 000 CellTox Green dyes were added for 1 hour prior to imaging. Images were obtained automatically with the ScanR acquisition software controlling a motorized Olympus IX-83 wide-field microscope, equipped with a Lumencor SpectraX light engine and Hamamatsu ORCA-FLASH 4.0, using an Olympus Universal Plan Super Apo 4×/0.16 AIR objective.

### Generation of GFP-G6PC3 and Flag-G6PC3 U2OS cells.

To generate the inducible U2OS cell line expressing G6PC3, the human G6PC3 cDNA in plasmid pcDNA3.1(+)-N-eGFP was synthesized from GenScript Biotech and recloned into pLVX-TetOn-Puro-GFP or pLVX-TetOn-Puro-Flag lentivirus vectors. HEK293T cells were transfected with 3 μg pLVX-GFP-G6PC3 or pLVX-Flag-G6PC3, 1 μg VSV-G (Clontech), and 1 μg PAX2 (Clontech) plasmids using JetPEI to produce lentivirus according to the manufacturer’s protocol. The culture media were changed 5 hours after transfection. Two days after transfection, the supernatant was collected by centrifugation at 300*g* for 5 minutes to obtain lentiviral particles. In total, 5 mL of the supernatant containing lentiviral particles was mixed with 5 mL of fresh U2OS medium containing polybrene (Sigma-Aldrich, H9268-5G) at 10 μg/mL and added to the U2OS cells. Two days after transduction, G6PC3 containing U2OS cells were selected using 3 μg/mL puromycin for 10 days. Subsequently, for the GFP-G6PC3 cell line, cells were induced with 1 μg/mL doxycycline for 2 days and sorted using FACS Aria III to ensure the presence of a moderate GFP-G6PC3–expressing population. The expression was verified by immunoblotting and immunostaining (Supplemental Figure 3, A and B).

### Bulk RNA-Seq sample preparation and sequencing.

For bulk RNA-Seq, the samples were divided into control group (siUNC) and experimental group (siG6PC3) with 3 biological replicates in each group. U2OS cells were transfected with 30 nM siRNA using Lipofectamine RNAiMAX (Thermo Fisher Scientific, 13778500). Cells were collected 48 hours after transfection following RNA extraction using AllPrep DNA/RNA Mini Kit (QIAGEN, 80204) on Qiacube MDX. Subsequently, RNA was subjected to DNase treatment with RNase-Free DNase Set (QIAGEN, 79254) and RNeasy Mini Kit (QIAGEN, 74106) on Qiacube MDX. cDNA was converted, and the libraries were pooled by Illumina Stranded Total RNA Prep with Ribo-Zero Plus kit (Illumina, 20040529) according to the manufacturer’s protocol. The sequencing was performed on Illumina NovaSeq 6000. The 150 bp paired-end RNA-Seq data were base-called and adapter-trimmed with bcl2fastq by the sequencing facility to generate the raw fastq files.

### RNA-Seq data QC and analysis.

For data processing, FastqQC (v0.11.9) (45) and FASTQ Screen (v0.15.0) (46) were used to evaluate the reads quality. The adapter-trimmed reads were further quality trimmed with Fastp (47) (v0.21.0) using default settings (‘--disable_adapter_trimming --correction --trim_poly_x --cut_tail --trim_front1 12 --trim_front2 12 --trim_tail1 1 --trim_tail2 1’). The trimmed reads were aligned to the hg38 genome assembly (canonical chromsomes only) using STAR (48) (v2.7.3a) in 2-pass mode and guided by a RefSeq (UCSC,2021/12/8) gene annotation (settings: --sjdbOverhang 133 --twopassMode Basic --outSAMtype BAM SortedByCoordinate --outSAMattributes All --outSAMunmapped Within --outFileNamePrefix siG6PC3R1 --outFilterMismatchNoverLmax 0.1 --outFilterMatchNmin 25 --outFilterMismatchNmax 10 --peOverlapNbasesMin 20). After mapping the reads were assigned to genes with featureCounts (49) (v1.5.1, settings: -p -B -C -s 2 -J) generating a count table. G6PC3 siRNA knockdown effect was validated by comparing G6PC3 normalized counts between control group and experimental group using DESeq2 (50) (v1.30.1) package in R (51) (v4.0.3). To check the distribution of count data between control group and experimental group, differential expression analysis (DEA) and statistical analysis was generated by DESeq2 (50) (v1.30.1) package in R (51) (v4.0.3).

### GSEA.

GSEA was performed using the clusterProfiler package (52) on KEGG database. The top 20 upregulated and top 20 downregulated pathways were obtained by ranking the normalized enrichment score (NES) values of GSEA results with *q* values less than 0.1, followed by plot in Prism software. The GSEA heatmap was generated based on gene expression z scores of genes in the HR repair pathway, while the KEGG pathway figure was based on the log_2_FC values.

### Analysis of TCGA BRCA cohort.

Data describing the clinical characteristics of patients were downloaded from cbioportal (53) (https://www.cbioportal.org/study/summary?id=brca_tcga). Small-scale somatic mutations in 1,097 patients, including point mutations, insertions and deletions, detected by whole-exome sequencing (WES) were downloaded from cbioportal (53) (https://www.cbioportal.org/study/summary?id=brca_tcga). Somatic variants affecting BRCA1, BRCA2, or G6PC3 were collected; however, we did not find any small-scale somatic alterations in G6PC3. Mutations found in BRCA1, and BRCA2 genes were annotated by InterVar (54); missense variants were also classified by AlphaMissense (55). Pathogenic germline variants, including pathogenic, likely pathogenic and prioritized variants of uncertain significance (VUS), were collected from ref. 56. We did not find any pathogenic variants affecting G6PC3. Putative copy number calls in 1,080 patients determined by GISTIC 2.0 were downloaded from cbioportal (53) (https://www.cbioportal.org/study/summary?id=brca_tcga). GISTIC 2.0 values correspond to the following copy number states: –2 = homozygous deletion; –1 = hemizygous deletion; 0 = neutral/no change; 1 = gain; and 2 = high-level amplification. Methylation (HM450) β-values for genes in 885 patients were downloaded from cbioportal (53) (https://www.cbioportal.org/study/summary?id=brca_tcga). For genes with multiple methylation probes, the β-value of the probe that is most anticorrelated with expression is considered. Expression z scores of tumor samples compared with the expression distribution of all log-transformed mRNA expression of adjacent normal samples (57) were downloaded from cbioportal (53) (https://www.cbioportal.org/study/summary?id=brca_tcga_pan_can_atlas_2018).

### BRCA1/2 deficiency status.

Patients with pathogenic germline variants or pathogenic somatic variants classified by InterVar or AlphaMissense with an accompanying loss-of-heterozygosity event detected by GISTIC 2.0 (hemizygous deletion) and patients with homozygous deletions of BRCA1 or BRCA2 were considered BRCA1/2 deficient. In addition, patients with BRCA1 methylation β-value ≥ 0.25 and expression z score ≤ –2 were labeled as BRCA1 deficient. Altogether, we identified 35 BRCA1- and 33 BRCA2-deficient patients. The remaining samples were labeled as BRCA1/2-intact patients.

### G6PC3 expression status.

Expression z score threshold of ± 2 was used to group each sample into 1 of 3 categories: low expression (z score ≤ –2), normal expression (–2 < z score < 2), high expression (z score ≥ 2).

### BRCA hormone receptor subtypes.

Data describing the clinical characteristics of patients were used to determine the hormone receptor subtype of patients based on IH: estrogen receptor (ER)-positive (ER_STATUS_BY_IHC == “Positive”), epidermal growth factor receptor 2^-^ (HER2^-^) (IHC_HER2 == “Negative”), and triple-negative (ER_STATUS_BY_IHC == “Negative” & PR_STATUS_BY_IHC == “Negative” & IHC_HER2 == “Negative”).

### Survival analysis.

The Kaplan-Meier method was used to estimate the survival curves, and multivariate models of overall survival were performed using Cox proportional hazards models accounting for sex, age, race, and primary tumor stage using the survival and survminer R packages.

### qPCR.

Total RNA was extracted using RNeasy Mini Kit (QIAGEN, 74106) according to the manufacturer’s instruction. Next, cDNA was synthesized using 500 ng of total RNA with RT2 First Strand Kit (Qiagen, 330404), and 12.5ng of cDNA was amplified using Maxima SYBR Green/ROX qPCR Master Mix (Thermo Fisher Scientific, K0221) on a LightCycler 480 (Roche). Relative mRNA expression was calculated using the ΔΔCt method with GAPDH as reference gene. qPCR primers used in this study are listed in Supplemental Table 4.

### Immunoblotting.

For immunoblotting, cells were lysed in RIPA buffer (Sigma-Aldrich, R0278) supplemented with protease inhibitor cocktail (Sigma-Aldrich, 5056489001), phosphatase inhibitor cocktail (Sigma-Aldrich, 4906845001), and 1 mM dithiothreitol (DTT). Benzonase (MilliporeSigma, 70746-3) at 1:1,000 dilution digestion to degrade DNA and RNA was then performed for 30 minutes on ice, following 10 minutes of centrifugation at 20,000*g* at 4°C. Protein Assay Dye Reagent Concentrate (Bio-Rad) was used to measure the protein concentration, and samples were diluted with 4× LDS Sample Buffer (Thermo Fisher Scientific, NP0007). Samples were further denatured at 55°C for 10 minutes. The denatured sample was separated using precast NuPAGE Bis-Tris 4-12% Gels (Thermo Fisher Scientific, NP0335BOX) in MOPS buffer (Thermo Scientific Fisher, NP0001) and blotted on to nitrocellulose membranes (GE Lifesciences). Membranes were incubated with blocking buffer (5 % blocking milk in PBST) for 1 hour at room temperature. The primary antibody was diluted in blocking buffer, and the membranes were incubated with the primary antibody overnight at 4°C. Furthermore, the membranes were washed 3 times for 10 minutes each with PBST and incubated with secondary HRP-conjugated antibody for 1 hour at room temperature. Finally, the membranes were washed 3 times for 10 minutes each in PBST and incubated with Immobilon Classico/Crescendo Western HRP substrate (MilliporeSigma) for 5 minutes. Chemiluminescence was detected using ChemiDoc Imaging System (Bio-Rad). Immunoblotting antibodies used in study are listed in Supplemental Table 7.

### CRISPR-Select assay.

CRISPR-Select assay was performed as described in ref. 34. Briefly, an iCas9-U2OS clonal cell line was developed using Cas9 expressed from a stably integrated TRE3G Edit-R Inducible Lentiviral Cas9 construct (Horizon, CAS11229) and validated for Cas9 expression. CRISPR-Select cassettes were designed by selecting gRNAs using the Sanger Institute Genome Editing (WGE) online tool. gRNAs were chosen to ensure the mutations targeted were within the seed region (PAM or 1-10 PAM-proximal nucleotides) to disrupt the Cas9 target site and maximize knock-in efficiency. The ssODN repair templates were designed to position the synonymous WT′ mutation within 1–3 nucleotides of the variant of interest to facilitate knock-in at similar frequencies. The Human Splicing Finder tool (http://www.umd.be/HSF3/) was used to confirm that WT′ mutations did not create splice sites, and the Codon Usage Database (http://www.kazusa.or.jp/codon) ensured that the mutations did not introduce rarely used codons. ssODN homology arms were 45 nucleotides long to get good knock-in efficiency.

gRNAs were used in the form of crRNA:tracrRNA duplexes (IDT) and reconstituted in nuclease-free duplex buffer at 10 or 100 μM. ssODNs were obtained as Ultramer DNA oligonucleotides at 100 μM in IDTE (pH 8.0). Cas9 expression was induced by 1 μg/mL doxycycline 24 hours before transfection. For transfection in a 6 cm dish, 150 pmol each of crRNA, and tracrRNA were incubated for 10 minutes at room temperature, followed by the addition of 250 μL OptiMEM and 20 pmol of variant and WT′ ssODNs. This nucleotide solution was mixed with 15 μL Lipofectamine RNAiMAX in 250 μL OptiMEM, incubated for 10 minutes, and then added to the iCas9-U2OS cells in fresh medium with doxycycline. On day 2 after CRISPR-Select delivery, an aliquot of cells was collected for early variant/WT′ analysis, while the rest were replated at 50,000–70,000 cells per 6 cm dish in complete medium. On day 7, cells were trypsinized, and 50,000–100,000 cells were replated and cultured until collection on day 12. crRNAs, ssODN repair templates, and PCR primers used in this study are listed in Supplemental Tables 3, 5, and 6, respectively.

### CRISPR/Cas9 sgRNA design for in vivo study.

The CRISPR/Cas9 sgRNAs for in vivo study were chosen from 2 different libraries: sgBrca1 (5′– AAGTACATCCCTTCAACCTG –3′) was selected from Mouse CRISPR Knockout Pooled Library (Brie); sgCcdc108 (5′– CCGACCTCTAGAAGCGGTAA –3′) and sgG6pc3 (5′– GCCG¬CCCAACACTTGGTGAG –3′) were selected from Mouse Improved Genome-wide Knockout CRISPR Library v2 (YUSA).

### LV production for in vivo study.

Cloning of the nontargeting sgRNA (5′– TGATTGGGGGTCGTTCGCCA –3′) and sgRNAs targeting *Brca1*, *Ccdc108*, and *G6pc3* into the PLentiCre vector was performed as previously described (58). To generate this vector, Cre-T2A was inserted into the lentiGuide-Puro vector (Addgene plasmid 52963) between the EF-1α promoter and the puromycin resistance fragment. The vector was subsequently validated by Sanger sequencing. Concentrated stocks of VSV-G pseudotyped lentivirus were produced by transient cotransfection of 4 plasmids in 293T as described (59). Lentiviral titers were determined using the qPCR lentivirus titration kit from Applied Biological Materials (LV900).

### Generation of somatic mouse models.

To generate *Brca1*^–^, *Ccdc108*^–^, or *G6pc3*-deficient tumors in combination with the loss of *Trp53*, 8-week-old FVB/*Rosa26-Cas9;Trp53^fl/fl^* (60, 61) female mice, genotyped as previously described (62, 63), were intraductally injected as described (35, 64) (*n* = 35) with lentiviruses encoding s*gBrca1*, sg*Ccdc108*, or sg*G6pc3* in combination with Cre. In brief, 20 μL of high-titre lentiviruses were injected into the fourth and the third mammary glands by using a 34G needle. Lentiviral titres ranging from 2 × 10^8^ transfection units (TU) per mL were used. Mice were monitored 3 times per week for tumor development and sacrificed when reaching the humane endpoint.

### Histology on tumor pieces.

Tumors were formalin-fixed overnight and paraffin-embedded (FFPE) by routine procedures. H&E staining was performed as described (65). H&E slides were used to classify mammary tumor lesion types. They were reviewed by a comparative pathologist in a blinded manner.

### PCR amplification and TIDE analysis.

Amplification of *Brca1*-exon 10, *Ccdc108*-exon 4, and *G6pc3*-exon 4 was performed with specific primers spanning the target sites (Brca1 forward [Brca1-FW]: 5′-GGCTCTCTAAGGTGCCCG-3’; Brca1 reverse [Brca1-RV]: 5′-ACTTAGTAACCCCCGACCCC-3′; Ccdc108-FW: 5′-GGGAGGGTCTTCCTAAGCTG-3′; Ccdc108-RV: 5′-GGAGAAGGAAGACATAGGTCCC-3′; G6pc3-FW: 5′-GATTCCGCAATCTCCACAGC-3′; G6pc3-RV: 5′-ACAAGTGAAACAGGACAGGACC-3′) and 1 μg of DNA template using the Q5 high-fidelity PCR kit from New England Biolabs. Amplicons were purified using the Isolate II PCR and Gel kit (Bioline). PCR products were Sanger sequenced using the RV primer for *Brca1*, FW primer for *Ccdc108*, and FW primer for *G6pc3*. CRISPR/Cas9-induced editing efficacy was predicted and quantified with the TIDE algorithm as described (66) (http://tide.nki.nl).

### Statistics.

Statistical analyses were performed using Graphpad v.7 software, except for the mouse study, the data for which was analyzed using R and package survival. The analysis of mouse survival data was conducted separately for tumor-free survival (TFS) and overall survival (OS), employing the same approach in each case: a Cox proportional-hazards model was fitted to the response (either TFS or OS), using the group (*Brca1*, *Ccdc108*, and *G6pc3*, each compared with the reference group NT) as a covariate. Additionally, we present the survival curves for the 4 different groups. Other analysis details are provided in the figure legend for specific tests.

### Study approval.

The clinical study was previously approved by the Capital Region of Denmark (H-4-2010-050), the Danish Data Protection Agency (RH-2016-353, I-Suite no. 05097), and the DBCG (jr. no. DBCG-2013-15). Animal experiments were approved by the Animal Ethics Committees of the Netherlands Cancer Institute. Mice were bred and maintained in accordance with institutional, national, and European guidelines for Animal Care and Use.

### Data availability.

RNA-Seq data are archived in GEO (GSE279092). Values for all data points shown in graphs, and values behind any reported means are provided in the Supporting Data Values file. Additional information is available upon request from the corresponding author.

## Author contributions

Conceptualization was contributed by CSS, FCN, JJ, MR, XL, MF, and ZS. Methodology was contributed by XL, AMDS, J Börcsök, TG, J Benada, JT, MB, HVDG, JYS, RM, MR, FCN, and JVJ, JJ. Investigation was contributed by XL, AMDS, J Börcsök, TG, J Benada, JT, MB, JVJ, AT, LS, HVDG, JYS, and RM. Visualization was contributed by XL, AMDS, J Börcsök, TG, J Benada, JT, JVJ, and MB. Supervision was contributed by CSS, JJ, FCN, MR, JVJ, MF, and ZS. Writing original draft was contributed by XL, AMDS, and CSS. Editing was contributed by CSS, XL, AMDS, JJ, MB, MR, FCN, TG, J Benada, JVJ and J Börcsök.

## Supplementary Material

Supplemental data

Unedited blot and gel images

Supporting data values

## Version 1 04/22/2025

In-Press Preview

## Version 2 06/09/2025

Electronic publication

## References

[1] Sung H (2021). Global Cancer Statistics 2020: GLOBOCAN estimates of incidence and mortality worldwide for 36 cancers in 185 countries. CA Cancer J Clin.

[2] Beitsch PD (2019). Underdiagnosis of hereditary breast cancer: are genetic testing guidelines a tool or an obstacle?. J Clin Oncol.

[3] Bose M (2022). A catalog of curated breast cancer genes. Breast Cancer Res Treat.

[4] Sulkowski PL (2018). Krebs-cycle-deficient hereditary cancer syndromes are defined by defects in homologous-recombination DNA repair. Nat Genet.

[5] Qu J (2017). Phosphoglycerate mutase 1 regulates dNTP pool and promotes homologous recombination repair in cancer cells. ” J Cell Biol.

[6] Sizemore ST (2018). Pyruvate kinase M2 regulates homologous recombination-mediated DNA double-strand break repair. Cell Res.

[7] Gustafsson NMS (2018). Targeting PFKFB3 radiosensitizes cancer cells and suppresses homologous recombination. Nat Commun.

[8] Herr P (2015). A genome-wide IR-induced RAD51 foci RNAi screen identifies CDC73 involved in chromatin remodeling for DNA repair. Cell Discov.

[9] Adamson B (2012). A genome-wide homologous recombination screen identifies the RNA-binding protein RBMX as a component of the DNA-damage response. Nat Cell Biol.

[10] Shieh JJ (2003). A glucose-6-phosphate hydrolase, widely expressed outside the liver, can explain age-dependent resolution of hypoglycemia in glycogen storage disease type Ia. J Biol Chem.

[11] Guionie O (2003). Identification and characterisation of a new human glucose-6-phosphatase isoform. FEBS Lett.

[12] Banka S, Newman WG (2013). A clinical and molecular review of ubiquitous glucose-6-phosphatase deficiency caused by G6PC3 mutations. Orphanet J Rare Dis.

[13] McDermott DH (2010). Severe congenital neutropenia resulting from G6PC3 deficiency with increased neutrophil CXCR4 expression and myelokathexis. Blood.

[14] Xia J (2009). Prevalence of mutations in ELANE, GFI1, HAX1, SBDS, WAS and G6PC3 in patients with severe congenital neutropenia. Br J Haematol.

[15] Veiga-da-Cunha M (2019). Failure to eliminate a phosphorylated glucose analog leads to neutropenia in patients with G6PT and G6PC3 deficiency. Proc Natl Acad Sci U S A.

[16] Desplantes C (2014). Clinical spectrum and long-term follow-up of 14 cases with G6PC3 mutations from the French Severe Congenital Neutropenia Registry. Orphanet J Rare Dis.

[17] Maxwell KN (2015). Prevalence of mutations in a panel of breast cancer susceptibility genes in BRCA1/2-negative patients with early-onset breast cancer. Genet Med.

[18] Zarrizi R (2020). Germline RBBP8 variants associated with early-onset breast cancer compromise replication fork stability. J Clin Invest.

[19] Rahmanian N (2021). Recent advances in γH2AX biomarker-based genotoxicity assays: A marker of DNA damage and repair. DNA Repair (amst).

[20] Ye CJ (2019). Micronuclei and genome chaos: changing the system inheritance. Genes (Basel).

[21] Wei-Shan H (2019). Cell cycle regulation of condensin Smc4. Oncotarget.

[22] Ma RM (2019). The prognostic value of the expression of *SMC4* mRNA in breast cancer. Dis Markers.

[23] Huang T (2021). Changes of EGFR and SMC4 expressions in triple-negative breast cancer and their early diagnostic value. Gland Surg.

[24] Crowley LC (2016). Quantitation of apoptosis and necrosis by Annexin V binding, propidium iodide uptake, and flow cytometry. Cold Spring Harb Protoc.

[25] Vermes I (2000). Flow cytometry of apoptotic cell death. J Immunol Methods.

[26] Liu X, Erikson RL (2003). Polo-like kinase (Plk)1 depletion induces apoptosis in cancer cells. Proc Natl Acad Sci U S A.

[27] Luzhna L (2013). Micronuclei in genotoxicity assessment: from genetics to epigenetics and beyond. Front Genet.

[28] Olive PL, Banáth JP (2006). The comet assay: a method to measure DNA damage in individual cells. Nat Protoc.

[29] Chopra N (2020). Homologous recombination DNA repair deficiency and PARP inhibition activity in primary triple negative breast cancer. Nat Commun.

[30] Gunn A, Stark JM (2012). I-SceI-based assays to examine distinct repair outcomes of mammalian chromosomal double strand breaks. Methods Mol Biol.

[31] Maréchal A, Zou L (2015). RPA-coated single-stranded DNA as a platform for post-translational modifications in the DNA damage response. Cell Res.

[32] Brianese RC (2018). BRCA1 deficiency is a recurrent event in early-onset triple-negative breast cancer: a comprehensive analysis of germline mutations and somatic promoter methylation. Breast Cancer Res Treat.

[33] Scully R (2019). DNA double-strand break repair-pathway choice in somatic mammalian cells. Nat Rev Mol Cell Biol.

[34] Niu Y (2022). Multiparametric and accurate functional analysis of genetic sequence variants using CRISPR-Select. Nat Genet.

[35] Annunziato S (2016). Modeling invasive lobular breast carcinoma by CRISPR/Cas9-mediated somatic genome editing of the mammary gland. Genes Dev.

[36] Liu X (2007). Somatic loss of BRCA1 and p53 in mice induces mammary tumors with features of human BRCA1-mutated basal-like breast cancer. Proc Natl Acad Sci U S A.

[37] Feng Y (2018). Breast cancer development and progression: Risk factors, cancer stem cells, signaling pathways, genomics, and molecular pathogenesis. Genes Dis.

[38] Nielsen FC (2016). Hereditary breast and ovarian cancer: new genes in confined pathways. Nat Rev Cancer.

[39] Forey R (2020). Mec1 is activated at the onset of normal S phase by low-dNTP pools impeding DNA replication. Mol Cell.

[40] Técher H (2017). The impact of replication stress on replication dynamics and DNA damage in vertebrate cells. Nat Rev Genet.

[41] Chou JY (2015). Type I glycogen storage diseases: disorders of the glucose-6-phosphatase/glucose-6-phosphate transporter complexes. J Inherit Metab Dis.

[42] Menzel T (2011). A genetic screen identifies BRCA2 and PALB2 as key regulators of G2 checkpoint maintenance. EMBO Rep.

[43] Zhang XD (2010). The use of SSMD-based false discovery and false nondiscovery rates in genome-scale RNAi screens. J Biomol Screen.

[44] Pierce AJ (1999). XRCC3 promotes homology-directed repair of DNA damage in mammalian cells. Genes Dev.

[45] https://www.bioinformatics.babraham.ac.uk/projects/fastqc/.

[46] http://www.bioinformatics.babraham.ac.uk/projects/fastq_screen/.

[47] Chen S (2018). fastp: an ultra-fast all-in-one FASTQ preprocessor. Bioinformatics.

[48] Dobin A (2013). STAR: ultrafast universal RNA-Seq aligner. Bioinformatics.

[49] Liao Y (2014). featureCounts: an efficient general purpose program for assigning sequence reads to genomic features. Bioinformatics.

[50] Love MI (2014). Moderated estimation of fold change and dispersion for RNA-Seq data with DESeq2. Genome Biol.

[51] https://cran.r-project.org/doc/manuals/r-release/fullrefman.pdf.

[52] Yu G (2012). clusterProfiler: an R package for comparing biological themes among gene clusters. OMICS.

[53] Cerami E (2012). The cBio cancer genomics portal: an open platform for exploring multidimensional cancer genomics data. Cancer Discov.

[54] Li Q, Wang K (2017). InterVar: clinical interpretation of genetic variants by the 2015 ACMG-AMP guidelines. Am J Hum Genet.

[55] Cheng J (2023). Accurate proteome-wide missense variant effect prediction with AlphaMissense. Science.

[56] Huang KL (2018). Pathogenic germline variants in 10,389 adult cancers. Cell.

[57] Hoadley KA (2018). Cell-of-origin patterns dominate the molecular classification of 10,000 tumors from 33 types of cancer. Cell.

[58] Sanjana NE (2014). Improved vectors and genome-wide libraries for CRISPR screening. Nat Methods.

[59] Follenzi A (2000). Gene transfer by lentiviral vectors is limited by nuclear translocation and rescued by HIV-1 pol sequences. Nat Genet.

[60] Marino S (2000). Induction of medulloblastomas in p53-null mutant mice by somatic inactivation of Rb in the external granular layer cells of the cerebellum. Genes Dev.

[61] Platt RJ (2014). CRISPR-Cas9 knockin mice for genome editing and cancer modeling. Cell.

[62] Derksen PW (2006). Somatic inactivation of E-cadherin and p53 in mice leads to metastatic lobular mammary carcinoma through induction of anoikis resistance and angiogenesis. Cancer Cell.

[63] Zingg D (2022). Truncated FGFR2 is a clinically actionable oncogene in multiple cancers. Nature.

[64] Krause S (2013). Intraductal injection for localized drug delivery to the mouse mammary gland. J Vis Exp.

[65] Doornebal CW (2013). A preclinical mouse model of invasive lobular breast cancer metastasis. Cancer Res.

[66] Brinkman EK (2014). Easy quantitative assessment of genome editing by sequence trace decomposition. Nucleic Acids Res.

[67] Rueden CT (2017). ImageJ2: ImageJ for the next generation of scientific image data. BMC Bioinformatics.

