# Supplementary material for: G6PC3 promotes genome maintenance and is a candidate mammary tumor suppressor
Source: JCI Insight. 2025 Apr 22;10(11):e186747. doi: 10.1172/jci.insight.186747 (PMC12220951; doi:10.1172/jci.insight.186747)
Supplement: Supplemental data [file jciinsight-10-186747-s239.pdf]

Supplementary File for

G6PC3 promotes genome maintenance and is a candidate mammary tumor suppressor

Xin Li *et al.*

*Corresponding author. Email: claus.storgaard@bric.ku.dk

This PDF file includes:

Supplemental Figures, 1-6

Supplemental Tables, 1-9

Figure S1

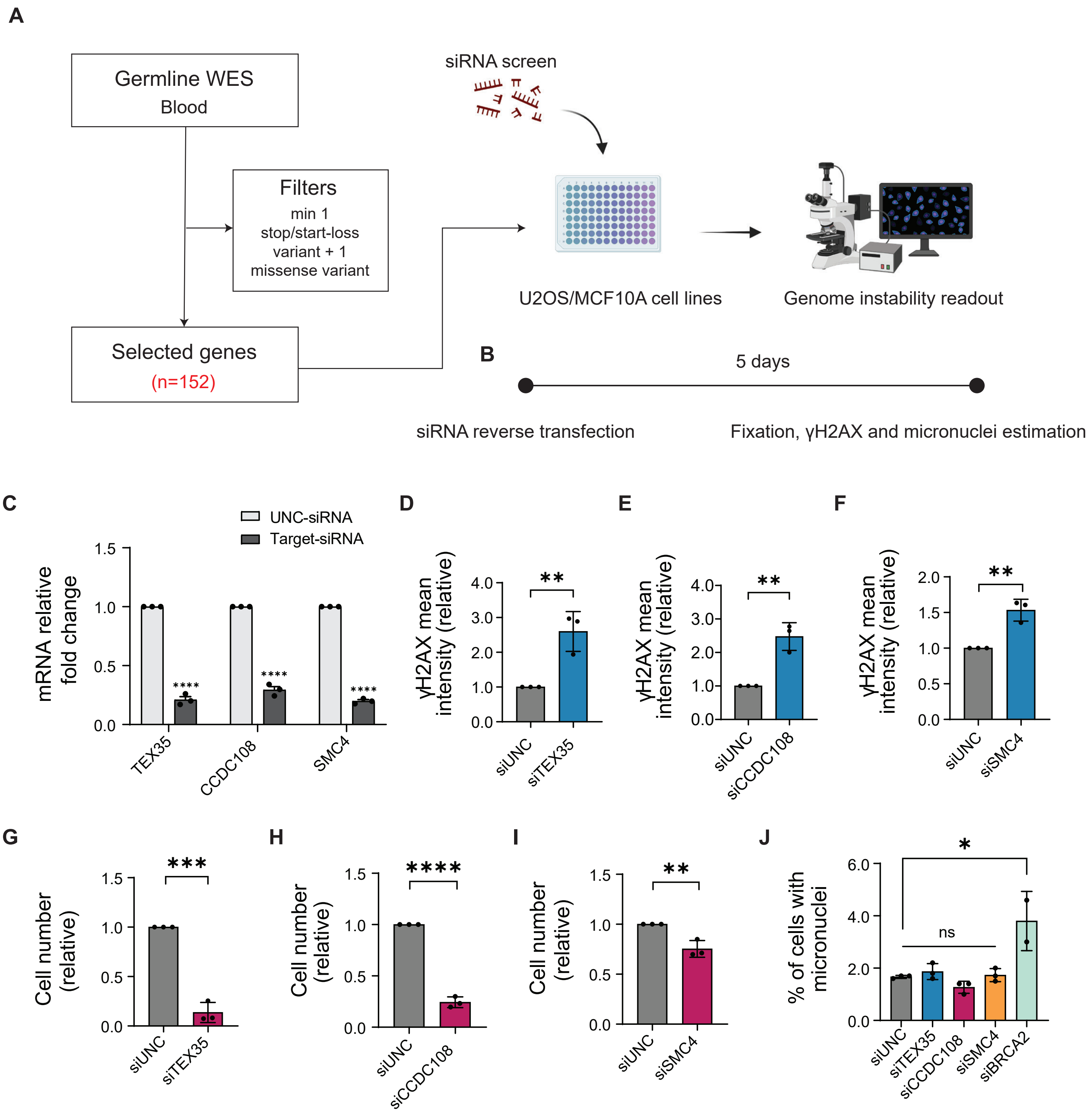

Supplementary Figure 1. Arrayed siRNA screen to identify genome maintenance factors. A. Schematic illustration of gene filtering and siRNA screening. **B.** Schematic representation of siRNA screen timeline. **C.** Efficiency of siRNA knockdown examined by qRT-PCR. U2OS cells were transfected with indicated siRNA (30 nM) for 48 h and mRNA were collected for analysis. **D - F.** Bar plot indicate relative γH2AX levels in U2OS cells treated with either TEX35 siRNA (D), CCDC108 siRNA (E) or SMC4 siRNA (F). Fold changes were normalized to control siRNA (set to 1). **G - I.** Bar plot indicate relative cell number in U2OS cells treated with either TEX35 siRNA (G), CCDC108 siRNA (H) or SMC4 siRNA (I). Fold changes were normalized to control siRNA (set to 1). **J.** Bar plot indicate percentage of U2OS cells with micronuclei, cells were treated with indicated siRNAs. Data are means ± s.d. from three biological replicates (two in panel J siBRCA2 condition); statistical significance of differences in C–I was assessed using two-tailed unpaired t-tests, while significance in J was determined using one-way ANOVA followed by Dunnett's test for multiple comparisons. *P<0.05, **P<0.01, ***P<0.001, ****P<0.0001, ns (not significant, P>0.05).

Figure S2

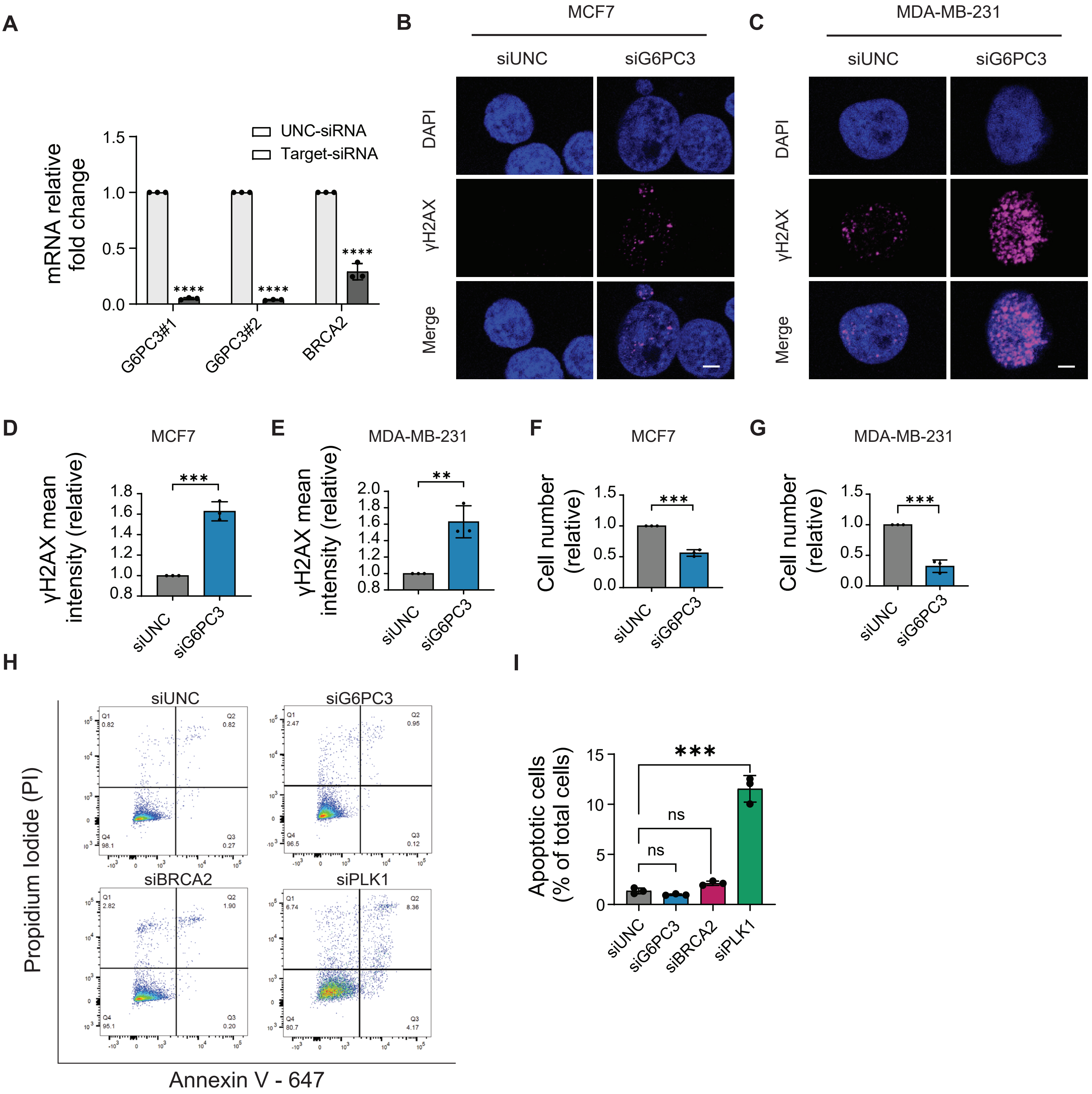

Supplementary Figure 2. G6PC3 is necessary to maintain genome stability in breast cancer cell lines. **A.** Efficiency of siRNA knockdown examined by qRT-PCR. U2OS cells were transfected with indicated siRNA (30 nM) for 48 h and mRNA were collected for analysis. **B & C.** Representative confocal images of DNA damage (γH2AX) in MCF7 (B) and MDA-MB-231 cell line (C), scale bar = 5 μm. **D & E.** QIBC analysis of γH2AX mean intensity in MCF7 (D) and MDA-MB-231 cell line (E). Cells were transfected with indicated siRNA (30 nM) and fold changes were normalized to control siRNA (set to 1). **F & G.** Bar plot indicate relative cell number in MCF7 (F) and MDA-MB-231 cell line (G). Fold changes were normalized to control siRNA (set to 1). **H.** Flow cytometry analysis of apoptosis induction using Annexin-V-647/PI. U2OS cells were transfected with indicated siRNA (30 nM) for 48 h. Early apoptotic and necrotic cells were represented by Annexin V + /PI – (lower right quadrant and Annexin-V + /PI + (upper right quadrant), respectively. **I.** Quantitative calculation of the proportion of apoptotic cells after siRNA treatment. Bar plot indicates means ± s.d. from three biological replicates; statistical significance of differences in A, D-G was evaluated using two-tailed unpaired t-tests, while significance in I was determined using one-way ANOVA followed by Dunnett's test for multiple comparisons.. $^{**}P<0.01$, $^{***}P<0.001$, $^{****}P<0.0001$, ns (not significant, $P>0.05$).

Figure S3

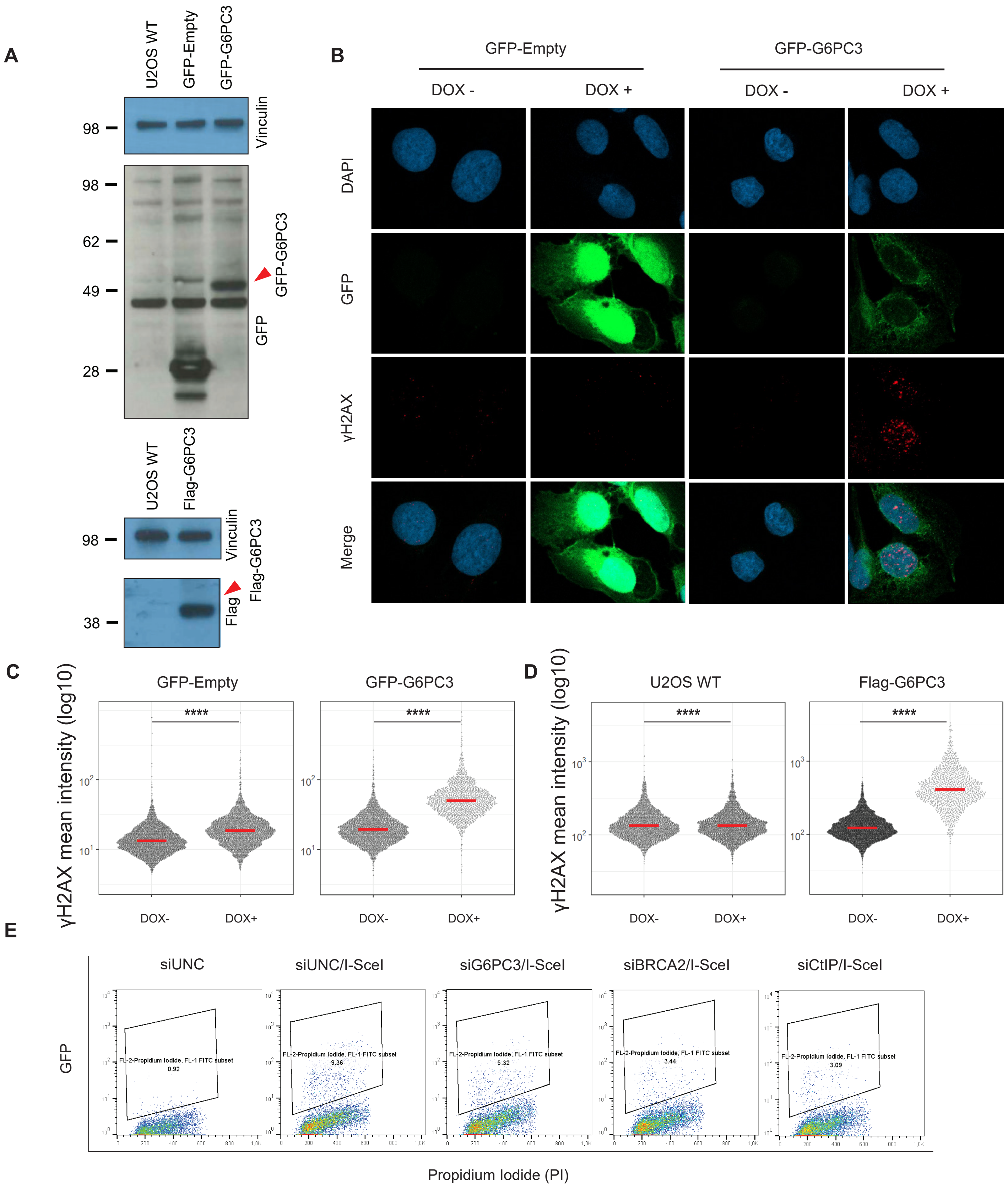

Supplementary Figure 3. Overexpression of G6PC3 activates DDR signaling. A. Representative Western blots for GFP and Flag expression. The expression was induced 48 hours before lysate collection by doxycycline (DOX). Endogenous vinculin was used as a loading control. **B.** Representative confocal images of G6PC3 localization and DNA damage (γH2AX) in U2OS cells. Empty-GFP or GFP-G6PC3 and γH2AX were visualized in the presence or absence DOX as indicated. Scale bar = 5 µm. **C & D.** γH2AX mean intensity quantified by QIBC in GFP-U2OS and GFP-G6PC3-U2OS cell lines (B), and WT-U2OS and Flag-G6PC3 cell lines (C). Representation of one out of three biological replicates, n>500. Statistical significance of differences was evaluated using two-tailed Mann-Whitney tests. **** *P*<0.0001. **E.** Representative flow cytometry images of different siRNA treatments in U2OS cells. Green fluorescence reports efficient HR repair, 10,000 events were analyzed.

Figure S4

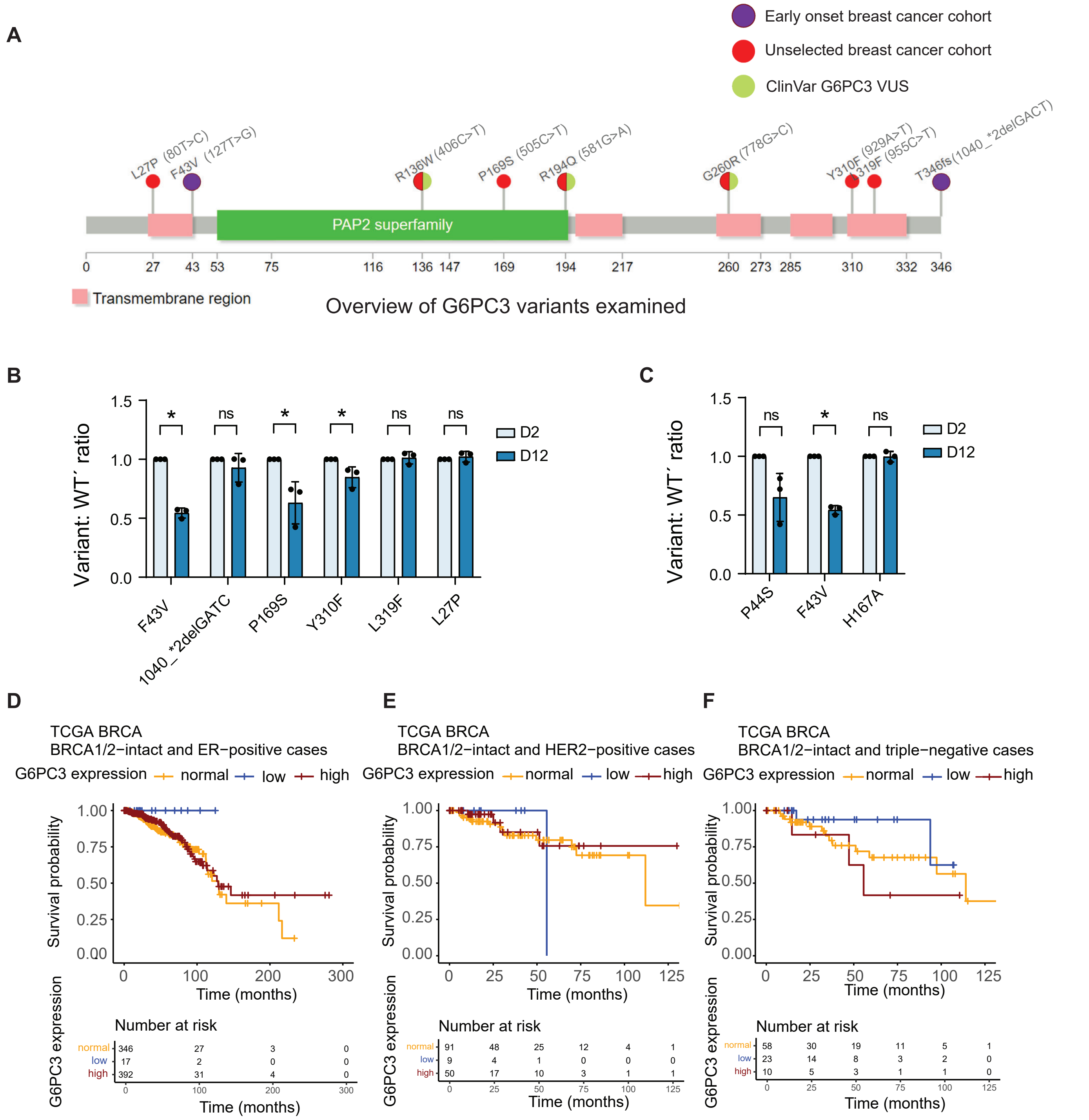

Supplementary Figure 4. Functional validation uncovers two potentially deleterious *G6PC3* variants. **A.** Overview of *G6PC3* variants examined. Purple dot indicates *G6PC3* variants identified in early onset breast cancer patients; red dot indicates *G6PC3* variants identified in unselected breast cancer patients; green dot indicates *G6PC3* variants co-observed in ClinVar database. Lollipop was plotted by http://www.bioinformatics.com.cn/srplot, an online platform for data analysis and visualization. **B.** CRISPR-Select analysis of *G6PC3* variants. ssODN cassettes were delivered to iCas9-U2OS cells and the ratios of Variant: WT´ were calculated on day-2 and day-12, and subsequently normalized to day-2 value. **C.** CRISPR-Select analysis of catalytic dead mutations in *G6PC3*. Bar plot indicates means ± s.d. from three biological replicates; statistical significance of differences was evaluated using two-tailed unpaired t-tests. *P<0.05, ns (not significant, P>0.05). **D - F.** Survival analysis of patients with breast cancer (BRCA) using The Cancer Genome Atlas (TCGA) BRCA cohort. Kaplan-Meier curves of overall survival (OS) stratified by G6PC3 expression status in the subset of BRCA1/2-intact and estrogen receptor (ER)-positive patients (D), epidermal growth factor receptor 2 (HER2)-positive patients (E) and triple-negative patients (F).

Figure S5

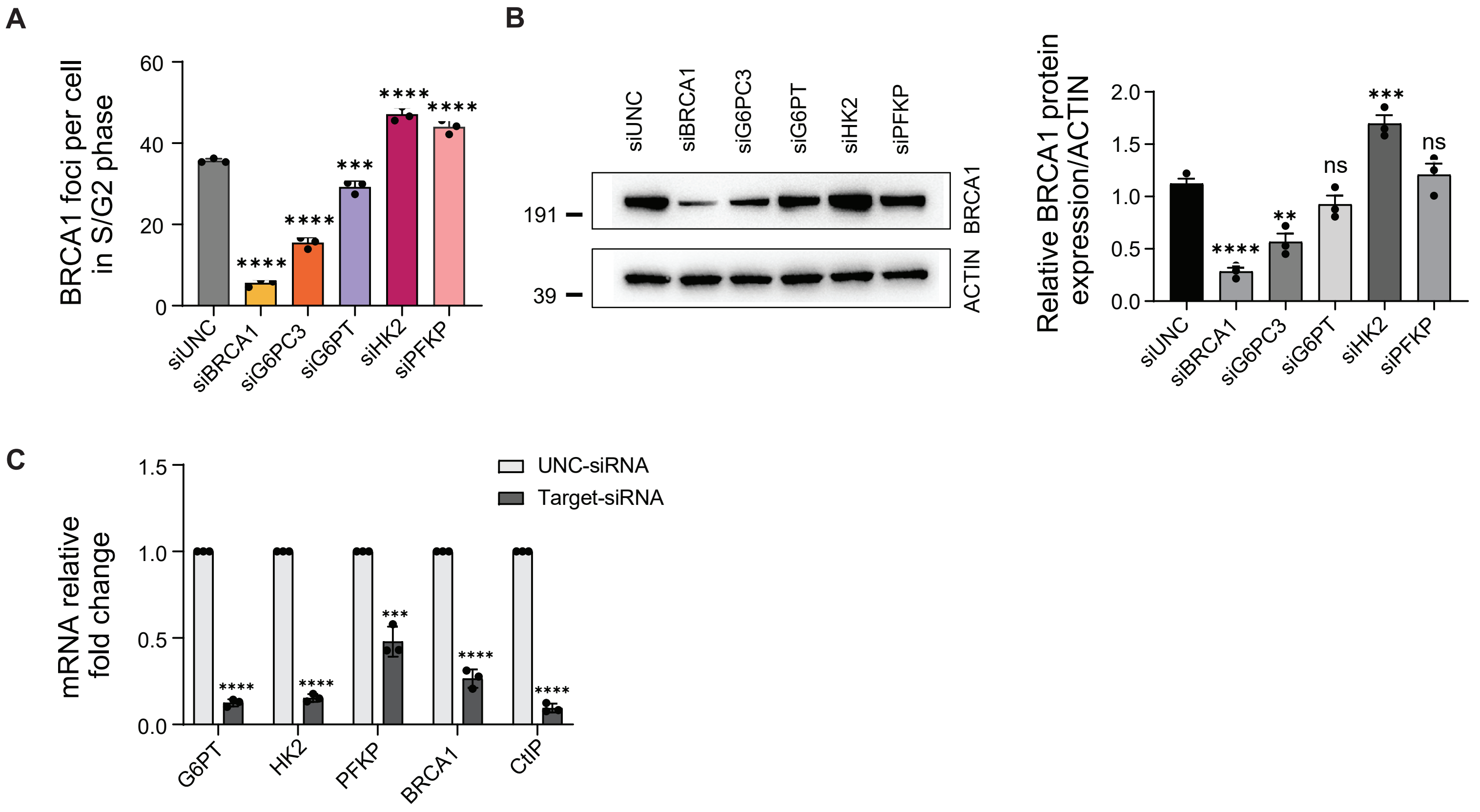

Supplementary Figure 5. G6PC3 catalytic activity has little impact on cell fitness. A. Changes in BRCA1 foci levels after depletion of different glycolytic genes under DNA damage. DNA DSBs were induced using 2 Gy IR and cells were fixed and imaged after 1 h. **B.** Changes in BRCA1 protein levels after depletion of different glycolytic genes depletion in U2OS cells. Endogenous ACTIN was used as a loading control. **C.** Efficiency of siRNA knockdown examined by qRT-PCR. U2OS cells were transfeScted with indicated siRNA (30 nM) for 48 h and mRNA were collected for analysis. Data are means ± s.d. of n=3 independent biological replicates; statistical significance of differences in A and B was evaluated using one-way ANOVA followed by Dunnett's test for multiple comparisons, while significance in C was determined using two-tailed unpaired t-tests. ***P*<0.01, ****P*<0.001, *****P*<0.0001, ns (not significant, *P*>0.05).

Figure S6

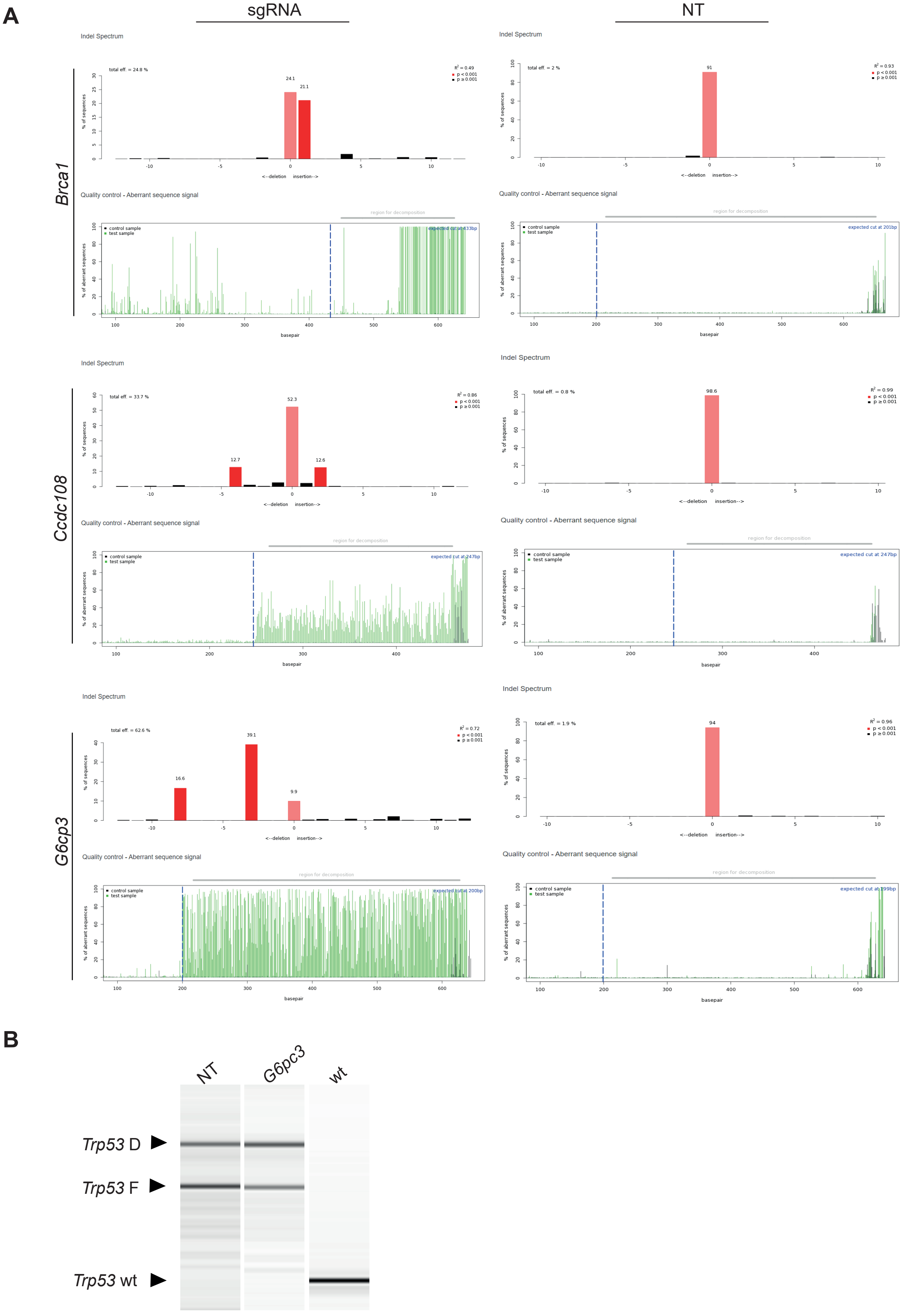

Supplementary Figure 6. Profiling of editing efficiency and tumor types in murine tumors. A. Representative TIDE analysis displaying the spectrum of indels of the targeted alleles in tumors: *Brca1* (mouse #2224639), *Ccdc108* (mouse #2227207), and *G6pc3* (mouse #2213085). NT tumors were compared to *Brca1*, *Ccdc108* (both mouse #2227222) and *G6pc3* (mouse #2312754) gRNAs. **B.** Representative *Trp53F/F* PCR in a sg*G6pc3*-injected tumor and a NT tumor. *Trp53* deletion (D) = 444 bp, and the Flox allele (F) = 353 bp band. The wild-type (wt) band (200bp) is observed at the control tumor (*Trp53*-proficient).

Supplemental Table 1. Full lists of arrayed siRNA screen.

U2OS Micronuclei

Gene, Symbol	Z-score	P-value
USP38	2.023590194	0.000210316
MYH4	1.857394547	0.000435303
SMC4	2.056792515	0.000628119
TEX35	1.179842447	0.001055109
G6PC3	2.287457272	0.002874189
RYR1	1.41125624	0.003831158
CENPH	1.478720404	0.003831714
IQCH	3.790284606	0.018434958
FLII	1.249999252	0.021158249
CSPP1	0.605872548	0.026461175
URM1	0.562734641	0.033564803
WDR87	2.476842438	0.034826301
MYH7	2.716311557	0.051583518
ABCB8	0.74256179	0.058189814
MAN2A2	0.598334257	0.058805825
WDR17	1.815396682	0.067339051
MYOM2	0.129523703	0.068040971
MUC16	0.297904222	0.072846313
DNTTIP2	0.408509847	0.074925917
NHSL1	0.326942073	0.099032096
FCN1	0.267678248	0.125460678
OR4K14	0.348687252	0.126506756
TDRD12	0.079759943	0.128946571
ABCA6	-0.04059466	0.130067742
MAP3K19	0.002566794	0.144797295
NR3C1	0.158871868	0.152651992
FHOD1	0.576114994	0.166772888
SYNE1	0.158817321	0.166943344
OBSL1	0.8185817	0.194224991
OTOG	-0.055271257	0.216224375
TRPM5	0.00924425	0.236582706
COL11A2	0.106934226	0.247692538
OTOF	0.318510207	0.250335601
DYSF	-0.117425407	0.260426908
NDUFA11	-0.136751123	0.264751061
ARHGEF10	0.128746787	0.274413487
TFEB	0.066779035	0.289457408
PRDM15	0.022947118	0.365189616
TARBP2	-0.086596507	0.374865895
PCDHGA2	-0.074621744	0.389354116
DNAH7	-0.019798959	0.39784925
PCDHGA7	-0.206027329	0.399447109
FAM22D (NUTM2D)	-0.003685334	0.408392382
AGAP9	-0.070646003	0.416387712

ACE	0.101029504	0.416785555
C2orf74	0.016701931	0.422042516
GOLGA8H	0.076422755	0.43302762
FBLN2	-0.036289843	0.437706801
ALK	-0.132131611	0.439112759
UGCGL2	0.107357098	0.44179648
PPT2	-0.130929635	0.459314815
SCNN1D	-0.017785463	0.467250664
FNDC7	-0.264839995	0.467433231
SDR42E2	0.018488938	0.470798973
CCDC108	-0.207391888	0.490321291
CADPS	1.243518501	0.511409581
TBC1D4	-0.30821282	0.560389447
SELP	-0.170397231	0.571852858
MEGF11	-0.26441291	0.655207565
UBXN6	-0.379705668	0.663376766
ATF7IP2	-0.152344543	0.664978448
GPR151	-0.48163715	0.696243569
LSM11	-0.114291897	0.69672883
HEATR5A	-0.155061407	0.703602412
NEK9	-0.323021742	0.717392318
WFS1	-0.394267421	0.73084805
KIAA1751 (c1orf222)	-0.199647857	0.737832076
KIF24	-0.142795542	0.759598782
WWOX	-0.366590789	0.777424293
ZNF724P	-0.202862984	0.781661455
RALBP1	-0.382980869	0.789035669
MED25	-0.166478499	0.792275863
DAB2IP	-0.338296729	0.795736811
MICAL3	-0.255100024	0.800704909
TRANK1	-0.426265457	0.804447598
KIR2DL3	-0.259156968	0.806389877
HYDIN	-0.410400046	0.810047034
ATM	-0.099612956	0.811497074
C14orf80 (TEDC1)	-0.457012655	0.822630889
MAP4K1	-0.239060929	0.833840212
RAET1E	-0.29243184	0.838792004
RAET1L	-0.358805046	0.845644649
MPDZ	-0.316595499	0.866813239
FZD6	-0.293963937	0.867873747
DPCR1	-0.257215512	0.868474226
MXD3	-0.485556366	0.87157424
HBS1L	-0.404958716	0.873051455
C2CD3	-0.297055481	0.885658528
CAPN12	-0.244099934	0.885757809
CFI	-0.202738091	0.888453502
RASSF3	-0.313452989	0.89000925
FARP2	-0.293980039	0.89054973
C10orf112 (MALRD1)	-0.226110076	0.893051022
RNH1	-0.341962389	0.904592057

DST	-0.312721848	0.90998143
N4BP2L2	-0.337043571	0.928198601
HSPB9	-0.500501014	0.929811894
PRKD1	-0.607968733	0.932033209
SARS2	-0.32239985	0.936393176
LIMK2	-0.392862446	0.937362953
SSTR5	-0.380535151	0.946558355
PTPN13	-0.373987179	0.955845193
FAM46A	-0.594996064	0.955886186
UBE4A	-0.493309867	0.960555199
SLTM	-0.509869984	0.960555199
NRROS	-0.415293328	0.96358612
ALPK3	-0.532922011	0.964164023
SLC26A2	-0.331273718	0.97441435
SNTB1	-0.492474255	0.978921293
PAN2	-0.494695535	0.979260034
RASSF7	-0.368981304	0.979921818
LILRB2	-0.677915924	0.980245023
TTC19	-0.369078481	0.980876454
ZNF862	-0.56999151	0.988119792
MYH15	-0.509312245	0.990275714
PKN3	-0.394902437	0.990596918
GARNL3	-0.3440644	0.991060021
CRYBG3	-0.656756473	0.991644142
TOM1	-0.579835984	0.992580933
LAMA1	-0.354152757	0.992829904
ZNF717	-0.562070408	0.993418511
MEF2A	-0.472209923	0.995350747
PTPRN	-0.567348071	0.995743278
SSX2IP	-0.446066229	0.996744235
ANKRD31	-0.493988841	0.997523633
NPHP3	-0.27446694	1
MFSD10	-0.46542158	1
ABI3BP	-0.469900082	1
IL17REL	-0.47409491	1
ALG12	-0.480091599	1
ATP10A	-0.480613302	1
CLCA4	-0.481495141	1
EIF4ENIF1	-0.484428884	1
RNF213	-0.486305639	1
FAM161A	-0.489672539	1
IL31RA	-0.516643936	1
PHLPP2	-0.531519705	1
NEB	-0.535547579	1
ADRA2A	-0.558530235	1
ADAP1	-0.572853433	1
L1TD1	-0.58937927	1
MROH8	-0.616631564	1
IL17RC	-0.634024208	1
ZBED4	-0.657951119	1

CARD14	-0.71185535	1
MEFV	-0.760815243	1
OR4N5	-0.775023945	1
FAAH2	-0.793301882	1
RBBP8NL (C20orf151)	-0.417757478	1
C11orf40	N/A	N/A

Supplemental Table 1. Full lists of arrayed siRNA screen (continued).

U2OS γH2AX

Gene, Symbol	Z-score	P-value
SMC4	5.222171964	1.36631E-05
TEX35	0.364198387	0.000508584
USP38	1.9076017	0.000601513
CCDC108	1.024389996	0.000942356
G6PC3	2.718306462	0.00140332
DNTTIP2	3.267809259	0.002525955
OBSL1	1.766776371	0.003100747
WDR87	4.079894862	0.003148397
TFEB	-0.088967322	0.009019618
ABCB8	1.214734456	0.014809838
OTOF	0.64184546	0.017204158
URM1	0.102977349	0.040269954
WDR17	1.37584673	0.041731391
FHOD1	3.9562548	0.051438819
NR3C1	-0.133022949	0.062467554
RYR1	0.509472417	0.068623049
TARBP2	0.670581948	0.080725376
FLII	0.706396814	0.081738386
MED25	-0.127481463	0.08765484
CSPP1	0.109173018	0.099762492
CARD14	0.427736212	0.100744795
MYH4	0.721323209	0.102802586
SELP	-0.146629458	0.108126554
IL17REL	-0.204693238	0.109623104
PCDHGA2	-0.09653466	0.123542947
MEGF11	-0.216479644	0.135531839
ALK	-0.185550812	0.139478711
TDRD12	-0.070713511	0.140799057
MUC16	0.021803489	0.146718056
CENPH	0.062599535	0.147530427
RALBP1	-0.094619609	0.149196531
SYNE1	0.114391531	0.153292437
IQCH	2.737599614	0.15663436
C11orf40	4.875732311	0.163990329
SDR42E2	-0.103889748	0.178721406
SCNN1D	0.507479989	0.182306676
COL11A2	0.205359637	0.220781557
LSM11	-0.262776949	0.237675442
PPT2	0.131822511	0.24942153
KIR2DL3	0.124679135	0.284266824
PRDM15	-0.123256108	0.286627149
WFS1	0.104895162	0.291818818
PCDHGA7	-0.334082591	0.314960916
AGAP9	-0.123526587	0.330156471

MAN2A2	0.150093812	0.331975786
FNDC7	0.959463698	0.367748718
OTOG	-0.12261739	0.374016488
TRPM5	-0.151369098	0.379384621
PAN2	-0.3976566	0.397621959
UGCGL2	0.866643531	0.424848854
ZNF724P	-0.257810412	0.444452423
FCN1	-0.13309573	0.448766077
FBLN2	-0.104362635	0.475900259
PRKD1	-0.254154085	0.475935411
CADPS	0.781991696	0.478006571
HEATR5A	-0.155213401	0.494363877
GARNL3	-0.391481346	0.541640017
TBC1D4	-0.245527289	0.547747672
FAM46A	-0.326524156	0.562712208
MICAL3	-0.223236365	0.568593128
NDUFA11	-0.227691355	0.569851461
OR4K14	-0.288690666	0.591233391
C2orf74	-0.305822829	0.599020802
DST	-0.379317856	0.61938329
N4BP2L2	-0.391594108	0.630015707
ACE	-0.358696803	0.655872512
LILRB2	-0.380187178	0.662501756
ANKRD31	-0.396134347	0.667331334
IL17RC	-0.350420242	0.667509778
CLCA4	-0.172498017	0.672771575
ABCA6	-0.300200591	0.681565107
NHSL1	-0.320424363	0.694224803
ADAP1	-0.284770227	0.705069255
DYSF	-0.342079265	0.710527802
RAET1L	-0.418096585	0.719201561
RNH1	-0.357284085	0.728010872
HYDIN	-0.325344254	0.735194462
MROH8	-0.333855587	0.743363197
MYOM2	-0.349368782	0.744453571
FAM161A	-0.405183503	0.758993124
ARHGEF10	-0.323749797	0.759079151
MAP3K19	-0.255095657	0.765405418
NEK9	-0.278728967	0.773426669
LAMA1	-0.382574814	0.778841305
RASSF3	-0.399724527	0.779593679
KIF24	-0.358653662	0.785926943
C10orf112 (MALRD1)	-0.238136087	0.791165121
LIMK2	-0.364828644	0.79925618
FAM22D (NUTM2D)	-0.215736291	0.808458844
MAP4K1	-0.474333721	0.816381531
NRROS	-0.289627058	0.81957793
EIF4ENIF1	-0.40699896	0.827320698
ADRA2A	-0.395229603	0.837126244
CAPN12	-0.456406223	0.847304117

WWOX	-0.460411029	0.864251837
CFI	-0.443411036	0.883701343
UBXN6	-0.422918047	0.886792678
SSTR5	-0.468220109	0.889567007
GOLGA8H	-0.396898376	0.892284365
MFSD10	-0.492108437	0.897435477
DAB2IP	-0.499109442	0.899879613
DNAH7	-0.393099052	0.900930355
MXD3	-0.397866076	0.901139105
MEFV	-0.41330563	0.907845015
ATM	-0.386183404	0.923623396
SARS2	-0.236413955	0.928471637
C2CD3	-0.44881097	0.933640252
RAET1E	-0.407851077	0.93448891
ZNF862	-0.446714293	0.935928472
TOM1	-0.527583761	0.938732168
FAAH2	-0.517495994	0.960484096
KIAA1751 (c1orf222)	-0.241456643	0.965137368
PTPN13	-0.458921699	0.966920412
SLTM	-0.474836546	0.968659877
HBS1L	-0.436242032	0.969036815
ATF7IP2	-0.479231426	0.971080037
MYH15	-0.522648526	0.97210712
PTPRN	-0.511617781	0.973999114
ZBED4	-0.452272983	0.973999114
OR4N5	-0.477332651	0.97441435
SLC26A2	-0.396886772	0.984532236
DPCR1	-0.493007158	0.988884259
ATP10A	-0.578888132	0.991209551
FARP2	-0.478888722	0.992058465
C14orf80 (TEDC1)	-0.239032823	0.993418511
SSX2IP	-0.504192864	0.994365725
UBE4A	-0.548823524	0.995350747
FZD6	-0.482764277	0.995667324
TRANK1	-0.447291697	0.995743278
RASSF7	-0.51531462	0.996859836
RNF213	-0.483778516	0.997430743
HSPB9	-0.500907443	0.997657185
RBBP8NL (C20orf151)	-0.25838313	0.997824957
ALG12	-0.625683406	0.998019133
SNTB1	-0.592894558	0.998510303
GPR151	-0.515757783	0.998698905
NEB	-0.592912123	0.998842913
ALPK3	-0.504398367	0.999192201
ABI3BP	-0.615658308	1
CRYBG3	-0.583988333	1
IL31RA	-0.539590024	1
L1TD1	-0.599239233	1
MEF2A	-0.577001492	1
MPDZ	-0.514587508	1

NPHP3	-0.539890451	1
PHLPP2	-0.59578008	1
PKN3	-0.562545598	1
TTC19	-0.577967071	1
ZNF717	-0.615629094	1
MYH7	N/A	N/A

Supplemental Table 1. Full lists of arrayed siRNA screen (continued).

MCF10A Micronuclei

Gene, Symbol	Z-score	P-value
SMC4	3.993200457	4.39843E-06
CENPH	2.597831073	0.000470832
ALK	3.51555258	0.000590855
WDR17	5.155386594	0.000779065
MAP4K1	1.346045726	0.010198722
C11orf40	2.177379638	0.017467844
TARBP2	1.023725871	0.020831566
MICAL3	1.131652053	0.021185975
MYH15	1.513361788	0.021739988
G6PC3	3.327790689	0.022303504
ZNF717	1.50337013	0.024483792
HSPB9	1.656099478	0.029736664
MEGF11	1.601834797	0.030213107
MPDZ	0.610832562	0.036601409
CLCA4	1.106231031	0.037312522
MAP3K19	1.408751524	0.039721499
CCDC108	1.462028424	0.045746093
UGCGL2	1.304368723	0.055089522
OTOG	0.825335072	0.089886645
FCN1	0.804876119	0.107486637
OR4K14	0.552297312	0.120497834
C10orf112 (MALRD1)	0.805257711	0.127950801
IQCH	0.399672262	0.129674545
FAM22D (NUTM2D)	0.89754061	0.137678868
ATM	0.825668158	0.148282571
PKN3	0.707914598	0.161525073
OR4N5	0.755838331	0.164916002
TRANK1	0.333717878	0.170902556
COL11A2	0.346358772	0.171003754
MEF2A	0.217458957	0.171069802
FARP2	0.596627953	0.178412116
IL31RA	0.572918194	0.184356168
OBSL1	0.552826435	0.189233127
HYDIN	0.276075616	0.190345774
ARHGEF10	0.78565678	0.193455666
ACE	0.114021492	0.197656874
CARD14	0.217220817	0.19951519
ATF7IP2	0.07123881	0.203016462
ATP10A	0.370779889	0.207965111
WDR87	-0.075936668	0.21925711
ALG12	0.353624975	0.224067237
MYH4	0.281385235	0.226025131
FBLN2	-0.154767012	0.234697651
TDRD12	0.25293717	0.23557077

FHOD1	0.480599161	0.242011075
SSTR5	0.263969639	0.24505509
GPR151	0.257692258	0.25329371
C2CD3	0.573159726	0.255834296
MUC16	0.253897807	0.27736541
MXD3	0.005318222	0.277934141
FNDC7	0.104290989	0.27877151
MFSD10	0.359108076	0.280492392
PPT2	0.435005259	0.292300975
SLTM	0.388280379	0.299598808
CAPN12	0.205717481	0.313468565
CSPP1	0.589477368	0.337183245
DYSF	0.015042897	0.374520894
ANKRD31	-0.052961291	0.423116335
KIF24	0.222491533	0.439850621
WWOX	-0.042138208	0.469865162
NEK9	-0.400180297	0.503055485
GARNL3	-0.414831286	0.505170401
LAMA1	-0.159939047	0.509034051
ADAP1	-0.555928077	0.518267861
HBS1L	-0.371642155	0.520880132
NHSL1	0.073051542	0.52188014
FZD6	-0.229062492	0.544943183
SELP	-0.112005089	0.551375669
ABCA6	-0.008535072	0.570802627
PHLPP2	-0.058917141	0.578633654
ZNF724P	-0.258759484	0.609200814
MAN2A2	-0.59035429	0.628167107
PTPN13	-0.271235368	0.642628681
TTC19	-0.032778317	0.649634191
DAB2IP	-0.357618331	0.706595491
ZBED4	-0.2790518	0.709466061
CFI	0.020226959	0.731433429
RAET1E	-0.470969012	0.756574529
SLC26A2	-0.453392384	0.779395363
C2orf74	0.111082419	0.796141942
ADRA2A	-0.412005901	0.815362836
SYNE1	-0.639262239	0.815362836
FAAH2	-0.130110517	0.817186868
SDR42E2	-0.621209586	0.832852651
PCDHGA2	-0.076219793	0.837786906
LIMK2	-0.614410938	0.837786906
MYOM2	-0.24369921	0.847248474
RYR1	-0.844154178	0.850285943
FLII	-0.614214898	0.853267053
PRKD1	-0.391501914	0.856192866
PTPRN	-0.42838416	0.859064422
NR3C1	-0.794558899	0.860480171
RNH1	-0.57123699	0.868702046
RASSF3	-0.615055799	0.870028044

ABI3BP	-0.214139219	0.878970571
CADPS	-0.33473493	0.881419782
OTOF	-0.242580074	0.893000364
N4BP2L2	-0.463313647	0.900483771
NDUFA11	-0.089481778	0.908453776
RAET1L	-0.202673459	1
MEFV	-0.287255274	1
KIR2DL3	-0.428294649	1
RALBP1	-0.444651255	1
EIF4ENIF1	-0.477354659	1
IL17REL	-0.5203132	1
DPCR1	-0.523858808	1
LILRB2	-0.52560918	1
HEATR5A	-0.576129715	1
TRPM5	-0.597919617	1
FAM46A	-0.615615863	1
SSX2IP	-0.621240955	1
UBE4A	-0.635980754	1
USP38	-0.645895845	1
UBXN6	-0.652313746	1
RNF213	-0.675872162	1
TBC1D4	-0.679547377	1
FAM161A	-0.711718641	1
AGAP9	-0.720491495	1
DST	-0.74452284	1
ALPK3	-0.753520108	1
DNTTIP2	-0.767659528	1
NPHP3	-0.768649268	1
RASSF7	-0.801565723	1
TOM1	-0.802937138	1
NRROS	-0.831433148	1
DNAH7	-0.862236576	1
PCDHGA7	-0.887054414	1
URM1	-0.891460213	1
L1TD1	-0.89978994	1
SNTB1	-0.903169776	1
NEB	-0.906368614	1
MED25	-0.919565891	1
SCNN1D	-0.924676548	1
PRDM15	-0.926580287	1
GOLGA8H	-0.9594134	1
CRYBG3	-0.979481752	1
PAN2	-1.001757795	1
WFS1	-1.010005665	1
ZNF862	-1.022696457	1
IL17RC	-1.027445668	1
LSM11	-1.05135685	1
MROH8	-1.113641755	1
MYH7	-1.116309412	1
TEX35	-1.307814691	1

ABCB8	-1.414224236	1
KIAA1751 (c1orf222)	-0.862642877	1
SARS2	-0.765548428	1
C14orf80 (TEDC1)	-0.700503336	1
RBBP8NL (C20orf151)	-0.925931023	1
TFEB	-0.502985259	1

Supplemental Table 1. Full lists of arrayed siRNA screen (continued).

MCF10A γH2AX

Gene, Symbol	Z-score	P-value
MYH7	1.998871345	0.000580353
SMC4	3.118060327	0.001303995
ALK	0.936356783	0.004311254
TEX35	2.219580723	0.005057235
WDR87	3.053939969	0.008116422
C11orf40	2.235518289	0.008759502
NR3C1	3.153002213	0.00990294
G6PC3	1.285498981	0.011360004
SYNE1	1.724339758	0.013733932
MED25	1.050764385	0.015588557
ABCB8	2.989567427	0.016387458
TBC1D4	0.773778738	0.016820679
PCDHGA7	1.263931596	0.017020876
UGCGL2	1.415126341	0.017600598
DNTTIP2	1.688125799	0.020707814
URM1	1.605079178	0.021227828
TFEB	0.580087811	0.021488666
CCDC108	1.448970573	0.024799377
MYH4	0.537863138	0.025564203
MUC16	1.708016203	0.031992362
MEGF11	0.849305452	0.055146666
SCNN1D	1.04799754	0.055366901
LAMA1	0.831612857	0.066813193
NHSL1	0.580856436	0.071846159
FHOD1	0.739544705	0.086573338
MFSD10	0.530114015	0.088694005
MROH8	0.818254771	0.094527822
PRKD1	0.688767079	0.097670395
DAB2IP	0.425468473	0.099737238
MAP3K19	0.809724534	0.102890177
MICAL3	0.282584943	0.106247874
OTOG	1.435275703	0.115891403
ARHGEF10	0.619976241	0.120369334
FAAH2	0.44328607	0.121683597
CSPP1	0.662237768	0.125633385
ADRA2A	0.565710529	0.126062278
FBLN2	0.721761301	0.158231379
OBSL1	0.145081642	0.172335887
COL11A2	0.254008196	0.189819789
FAM46A	0.16927369	0.201478219

FNDC7	-0.222997063	0.201770749
WDR17	0.98749142	0.207395881
PKN3	0.271826204	0.2092228
MAN2A2	1.17098066	0.212420644
DYSF	0.353182923	0.23450851
CARD14	0.436276592	0.235322623
IL31RA	-0.10066263	0.237101065
SLC26A2	0.228025672	0.248821991
FCN1	-0.136811497	0.251662898
CENPH	-0.215006412	0.252978772
C2CD3	-0.028755799	0.256711423
LILRB2	0.105617613	0.283340494
PCDHGA2	0.24540689	0.294440827
IQCH	0.277689966	0.296395801
RBBP8NL (C20orf151)	-0.280541517	0.307287112
HSPB9	0.078375555	0.335835867
RYR1	-0.080572412	0.340488551
SELP	0.229364898	0.348382751
C2orf74	0.493495955	0.359862508
TDRD12	0.068484	0.370257863
PRDM15	-0.101015816	0.377498862
RALBP1	-0.158432037	0.39386761
NRROS	-0.004643028	0.395525783
ZBED4	-0.121511611	0.401468818
USP38	0.255323039	0.402338089
MYH15	-0.034190832	0.412318298
TTC19	-0.257600802	0.422163325
DPCR1	-0.069152871	0.425132798
ATF7IP2	-0.380680762	0.449811359
FLII	-0.389862016	0.461539868
FAM22D (NUTM2D)	-0.178877181	0.462177879
KIF24	0.074865562	0.471968751
OR4K14	-0.127020512	0.480671548
CADPS	-0.241370897	0.528521503
NEK9	-0.312162337	0.537762671
PTPRN	-0.377756429	0.572670126
CAPN12	-0.456721857	0.579139341
SARS2	-0.294275961	0.589401939
GOLGA8H	-0.391229552	0.594837763
KIAA1751 (c1orf222)	-0.256001132	0.595283961
SDR42E2	-0.219605985	0.601368224
ATP10A	-0.327294413	0.61357302
GARNL3	-0.354528622	0.616468337
WWOX	-0.404838674	0.627019676
ABCA6	-0.625415037	0.631669156

PAN2	-0.512294464	0.642134196
CLCA4	-0.465570298	0.671076736
LIMK2	-0.649508807	0.677405228
ANKRD31	-0.507742127	0.678553403
WFS1	-0.429757554	0.68567482
CFI	-0.667346273	0.689018551
TARBP2	-0.552735891	0.693428377
MEFV	-0.478333396	0.697901443
MEF2A	-0.989140514	0.697901443
MXD3	-0.687121893	0.698788415
ALPK3	-0.365202195	0.716162934
MAP4K1	-0.446766724	0.723374672
ABI3BP	-0.459368472	0.734214997
ZNF724P	-0.472550329	0.745587934
PTPN13	-0.669758711	0.78346189
FARP2	-0.601758452	0.789061635
MPDZ	-0.672349808	0.789691512
ADAP1	-0.499171665	0.7956912
IL17REL	-0.605732099	0.829757203
HEATR5A	-0.76227239	0.840563851
IL17RC	-0.663126884	0.84258421
KIR2DL3	-0.653496202	0.855919524
ZNF717	-0.710105499	0.857010085
LSM11	-0.734113424	0.858476022
ALG12	-0.65870741	0.868720729
TRANK1	-0.725191435	0.870361051
N4BP2L2	-0.756369671	0.876771019
RASSF3	-0.782955762	0.880831021
NPHP3	-0.800669623	0.900912781
C10orf112 (MALRD1)	-0.545638435	0.901105311
HYDIN	-0.700106217	0.915168202
ATM	-0.811282932	0.921866078
NDUFA11	-0.70372712	0.926178054
GPR151	-0.876430249	0.942028184
L1TD1	-0.829815447	0.942298884
UBE4A	-0.9170505	0.946736632
ACE	-0.728023107	0.954363902
RNH1	-0.876601599	0.955387947
OR4N5	-0.849687709	0.955892382
TRPM5	-0.858274716	0.960669011
TOM1	-0.903778545	0.968140286
RAET1E	-0.934080835	0.969982749
UBXN6	-0.697143138	0.974968307
CRYBG3	-0.959990399	0.982162925
HBS1L	-0.826434217	0.984127003

ZNF862	-0.926265107	0.985523737
SSTR5	-0.839967913	1
OTOF	-0.928516	1
PPT2	-0.952732663	1
RAET1L	-0.969034749	1
DST	-0.973848867	1
MYOM2	-0.999589907	1
PHLPP2	-1.005783532	1
RNF213	-1.010366509	1
SLTM	-1.01378558	1
RASSF7	-1.023411132	1
DNAH7	-1.030707163	1
EIF4ENIF1	-1.031834723	1
FZD6	-1.036671781	1
FAM161A	-1.040319957	1
SSX2IP	-1.04545469	1
AGAP9	-1.046586884	1
SNTB1	-1.125398329	1
NEB	-1.147790576	1
C14orf80 (TEDC1)	-0.598848544	1

Supplemental Table 2. Sequence of siRNAs.

siRNA name	siRNA sequence 5' to 3'
siUNC	UAACGACGCGACGACGUAA
siBRCA1	AAGGAACCUGUCUCCACAAAG
siBRCA2	CCAACUUUGUCCUUAACUA
siCtIP	GCUAAAACAGGAACGAAU
siG6PC3#1	GGAUCAGCCUCAUCACCGA
siG6PC3#2	CCAAGAUCCUCUUUCUGUU
siG6PT	CGAAACAUCCGCACCAAGA
siHK2	CCTGGGTGAGATTGTCCGTAA
siPFKP	GGCUGAAGGAGCAAUUGAUTT
siPLK1	GCACAUACCGCCUGAGUCU

All siRNAs are H. sapiens specific.

Supplemental Table 3. Sequence of crRNAs.

crRNA name	crRNA sequence 5’ to 3’
gG6PC3	CTTTCTGTTCTACTTCCCCG
gBRCA1	GACGTCTGTCTACATTGAAT
gBRCA2	CCATTATTGAACTTACAGAT
G6PC3-F43V	CTTTCTGTTCTACTTCCCCG
G6PC3-1040_*2delGACT	ACACAAGAAGTCAGGAAGAG
G6PC3-P169S-crRNA	GGCCAGCCAGCACCTGGTGA
G6PC3-Y310F-crRNA	GGAAATTGAAAATGTAGAAG
G6PC3-L319F-crRNA	ATTTCCTCAAGTACACCCTC
G6PC3-L27P-crRNA	GGCTGGAGAACGTGTGGCTC

All crRNAs are H. sapiens specific.

Supplemental Table 4. Sequence of qPCR primers.

Primer name	Primer sequence
BRCA1_qPCRF	GAAACCGTGCCAAAAGACTTC
BRCA1_qPCRR	CCAAGGTTAGAGAGTTGGACAC
BRCA2_qPCRF	TCTGTTTCCACACCTGTCTC
BRCA2_qPCRR	CTACTCAAGAAATCCAAGGCTC
CtIP_qPCRF	CAGGAACGAATCTTAGATGCACA
CtIP_qPCRR	GCCTGCTCTTAACCGATCTTCT
G6PC3_qPCRF	CTTCTATGGGTTGACTGCAC
G6PC3_qPCRR	ACTTGAAGGCTAGGCTGATG
G6PT_qPCRF	CAGGGCTATGGCTATTATCGCAC
G6PT_qPCRR	ATGGCTCAAACCACTTCCGCAG
GAPDH_qPCRF	GGAGCGAGATCCCTCCAAAAT
GAPDH_qPCRR	GGCTGTTGTCATACTTCTCATGG
HK2_qPCRF	GAGCCACCACTCACCCTACT
HK2_qPCRR	CCAGGCATTCGGCAATGTG
PFKP_qPCRF	CCCATCACCTCTGAGAAAATC
PFKP_qPCRR	TTCAGTGACACGACGCAAG

Supplemental Table 5. Sequence of ssODN repair templates.

ssODN name	ssODN sequence
Reference sequence	TGGATCACCTTTCTGGGCGATCCCAAGATCCTCTTTCTGTTCTACTTCCCCGCGGCCTACTACGCCTCCCGCCGTGTGGGCATCGCGGTGCTC
G6PC3-F43V (WT´)	TGGATCACCTTTCTGGGCGATCCCAAGATCCTCTTTCTGTTCTACTTTCCCGCGGCCTACTACGCCTCCCGCCGTGTGGGCATCGCGGTGCTC
G6PC3-F43V (Variant)	TGGATCACCTTTCTGGGCGATCCCAAGATCCTCTTTCTGTTCTACGTCCCCGCGGCCTACTACGCCTCCCGCCGTGTGGGCATCGCGGTGCTC
Reference sequence	GTGCACATGTTCAGTGCCCAGGAAGCACCGCCCATCCACTCTTCCTGACTTCTTGTGTGCCTCCCTTTCCTTTCCCTCCCACAAAGCCAACAC
G6PC3-1040_*2delGACT (WT´)	GTGCACATGTTCAGTGCCCAGGAAGCACCGCCCATCCACTCTTCTTAACTTCTTGTGTGCCTCCCTTTCCTTTCCCTCCCACAAAGCCAACAC
G6PC3-1040_*2delGACT (Variant)	GTGCACATGTTCAGTGCCCAGGAAGCACCGCCCATCCACTCTTCTTCTTCTTGTGTGCCTCCCTTTCCTTTCCCTCCCACAAAGCCAACAC
Reference sequence	CTTTTGGCGGTTGGCTTGTCGCGAATCTTCATCTTAGCACATTTCCCTCACCAGGTGCTGGCTGGCCTAATAACTGGTGAGCAACTGGGGCAA
G6PC3-P169S (WT´)	CTTTTGGCGGTTGGCTTGTCGCGAATCTTCATCTTAGCACATTTCCCCCACCAGGTGCTGGCTGGCCTAATAACTGGTGAGCAACTGGGGCAA
G6PC3-P169S (Variant)	CTTTTGGCGGTTGGCTTGTCGCGAATCTTCATCTTAGCACATTTCTCTCACCAGGTGCTGGCTGGCCTAATAACTGGTGAGCAACTGGGGCAA
Reference sequence	TGCTGGGCCCCCTGGACTGGCTGGGCCACCCCCCTCAGATCAGCCTCTTCTACATTTTCAATTTCCTCAAGTACACCCTCTGGCCATGCCTAG
G6PC3-Y310F (WT´)	TGCTGGGCCCCCTGGACTGGCTGGGCCACCCCCCTCAGATCAGCCTCTTCTATATTTTCAATTTCCTCAAGTACACCCTCTGGCCATGCCTAG
G6PC3-Y310F (Variant)	TGCTGGGCCCCCTGGACTGGCTGGGCCACCCCCCTCAGATCAGCCTCTTCTTCATTTTCAATTTCCTCAAGTACACCCTCTGGCCATGCCTAG
Reference sequence	ACCCCCCTCAGATCAGCCTCTTCTACATTTTCAATTTCCTCAAGTACACCCTCTGGCCATGCCTAGTCCTGGCCCTCGTGCCCTGGGCAGTGC
G6PC3-L319F (WT´)	ACCCCCCTCAGATCAGCCTCTTCTACATTTTCAATTTCCTCAAGTACACCCTGTGGCCATGCCTAGTCCTGGCCCTCGTGCCCTGGGCAGTGC
G6PC3-L319F (Variant)	ACCCCCCTCAGATCAGCCTCTTCTACATTTTCAATTTCCTCAAGTACACCTTCTGGCCATGCCTAGTCCTGGCCCTCGTGCCCTGGGCAGTGC

Supplemental Table 5. Sequence of ssODN repair templates (continued).

ssODN name	ssODN sequence
Reference sequence	GGCGCTACAGAACCAGCTAGCCTGGCTGGAGAACGTGTGG**CTC**TGGATCACCTTTCTGGGCGATCCCAAGATCCTCTTTCTGTTCTACTTCCC
G6PC3-L27P (WT´)	GGCGCTACAGAACCAGCTAGCCTGGCTGGAGAACGTGTGG**CTG**TGGATCACCTTTCTGGGCGATCCCAAGATCCTCTTTCTGTTCTACTTCCC
G6PC3-L27P (Variant)	GGCGCTACAGAACCAGCTAGCCTGGCTGGAGAACGTGTGG**CCC**TGGATCACCTTTCTGGGCGATCCCAAGATCCTCTTTCTGTTCTACTTCCC

The codon affected by mutagenesis is in bold. WT´ nucleotide and amino acid substitutions are indicated in blue fond. Variant nucleotide and amino acid substitutions are indicated in red fond. The crRNA target site is underlined in the reference sequence. The PAM is underlined and indicated in purple fond in the reference sequence.

Supplemental Table 6. Sequence of PCR primers.

Primers used in first round PCR for amplicon sequencing

Primer name	Primer sequence
G6PC3-F43V-F	**ACACTCTTTCCCTACACGACGCTCTTCCGATCT**GACTCTGGTTTCCGCCCTG
G6PC3-F43V-R	**TGACTGGAGTTCAGACGTGTGCTCTTCCGATCT**GATCCAGAGCACCGCGATG
G6PC3-1040_*2delGACT-F	**ACACTCTTTCCCTACACGACGCTCTTCCGATCT**ACATGTTCAGTGCCCAGGAA
G6PC3-1040_*2delGACT-R	**TGACTGGAGTTCAGACGTGTGCTCTTCCGATCT**GGAGAGAGAAGGCCCATCTT
G6PC3-P169S-F	**ACACTCTTTCCCTACACGACGCTCTTCCGATCT**AGGAGGCCAAGCTGTGTATG
G6PC3-P169S-R	**TGACTGGAGTTCAGACGTGTGCTCTTCCGATCT**TGATCCTCCCACTGCCAAAG
G6PC3- Y310F -F	**ACACTCTTTCCCTACACGACGCTCTTCCGATCT**ACTCTCCCTGCTATGCCCA
G6PC3- Y310F -R	**TGACTGGAGTTCAGACGTGTGCTCTTCCGATCT**CACTGAACATGTGCACTGCC
G6PC3- L319F -F	**ACACTCTTTCCCTACACGACGCTCTTCCGATCT**GGGAAATGGCCAGAAGATAGC
G6PC3- L319F -R	**TGACTGGAGTTCAGACGTGTGCTCTTCCGATCT**GTCAGGAAGAGTGGATGGGC
G6PC3- L27P -F	**ACACTCTTTCCCTACACGACGCTCTTCCGATCT**GCTTCGTTGCCTGGACTCT
G6PC3- L27P -R	**TGACTGGAGTTCAGACGTGTGCTCTTCCGATCT**GATCCAGAGCACCGCGATG

Supplemental Table 7. Antibodies used for Immunoblotting.

Target	Dilution	Manufacturer	Calatog Nr.	Species
BRCA1	1:300	Santa Cruz	SC6954	Mouse
Actin	1:150000	Millipore	MAB1501	Mouse
Vinculin	1:10000	Sigma	V9131	Mouse

Supplemental Table 8. Profiling of editing efficiency in murine tumors.

Gene	#Mouse	TIDE total effect (%)
Brca1	2218977	21
	2218966	1.7
	2218975	3.9
	2218965	12.6
	2218974	9.7
	2224640	1.8
	2224639	24.8
NT	2227220	3
	2312754	4.2
	2227222	2
Ccdc108	2228208	1.7
	2227207	33.7
	2228206	8.2
	2238034	6.7
	2234645	7
	2234646	5.7
	2240123	5.8
	2255823	6.1
	2255824	17.3
	2255825	1.2
	2255826	1.4
NT	2227220	6.1
	2312754	1.6
	2227222	0.8
G6pc3	2211174	28.3
	2211175	14.5
	2213085	62.6
	2213086	34.3
	2312751	6.6
	2312753	6.6
	2312750	6.7
NT	2227220	5.8
	2312754	1.9
	2227222	4.3

TIDE total effect values (%) of the main tumor of each mice injected with sgNT (n=3), sg*Brca1* (n=7), sg*Ccdc108* (n=11), or sg*G6pc3* (n=7). NT tumors were compared to *Brca1*, *Ccdc108* and *G6pc3* gRNAs.

Supplemental Table 9. Profiling of tumor types in murine tumors.

Gene	#Mouse	Classification
NT	2227220	sarcoma
	2312754	sarcoma
	2227222	sarcoma
Brca1	2218977	sarcoma
	2218966	sarcoma
	2218975	adenocarcinoma
	2218965	sarcoma
	2218974	adenocarcinoma
	2224640	sarcoma
	2224639	sarcoma
Ccdc108	2228208	squamous cell carcinoma - sarcoma
	2227207	sarcoma
	2228206	sarcoma
	2238034	sarcoma
	2234645	sarcoma - adenocarcinoma
	2240123	sarcoma - adenocarcinoma - squamous differentiation
	2255823	sarcoma
	2255824	sarcoma
	2255825	sarcoma
	2255826	sarcoma - adenocarcinoma
G6pc3	2211174	sarcoma
	2211175	sarcoma
	2213085	sarcoma
	2213086	sarcoma - adenocarcinoma
	2312751	sarcoma
	2312753	sarcoma - adenocarcinoma
	2312750	sarcoma

Tumor type scoring of the main tumor of each mouse injected with NT (n=3), sg*Brca1* (n=7), sg*Ccdc108* 1041 (n=10) or sg*G6pc3* (n=7).
