# Supplementary material for: G6PC3 promotes genome maintenance and is a candidate mammary tumor suppressor
Source: JCI Insight. 2025 Apr 22;10(11):e186747. doi: 10.1172/jci.insight.186747 (PMC12220951; doi:10.1172/jci.insight.186747)
Supplement: Unedited blot and gel images [file jciinsight-10-186747-s240.pdf]

Blot and gel images

1. Full unedited gel for Figure 4A (from ChemiDoc Imager)

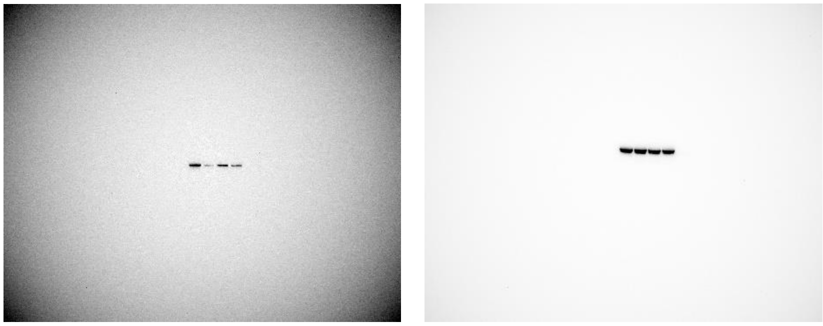

2. Full unedited gel for Figure S3A

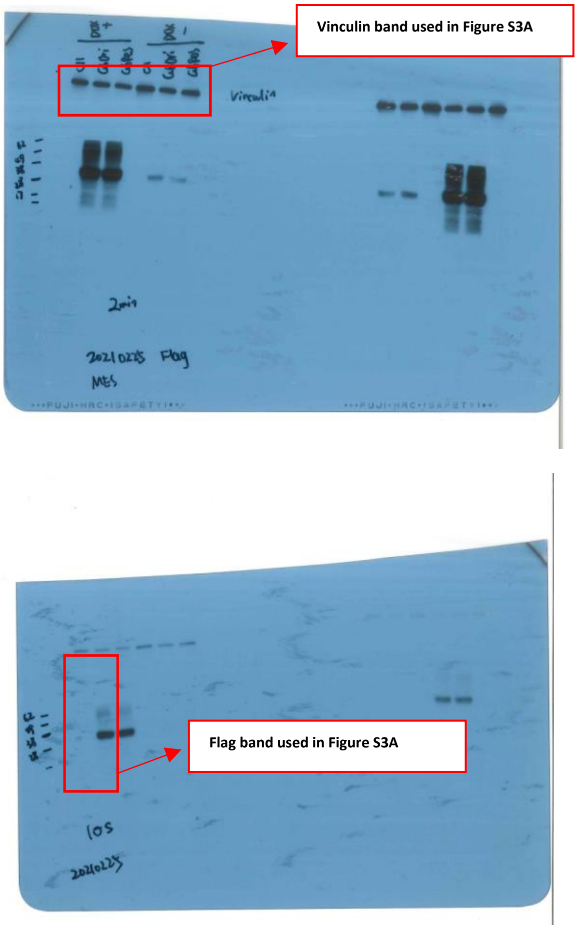

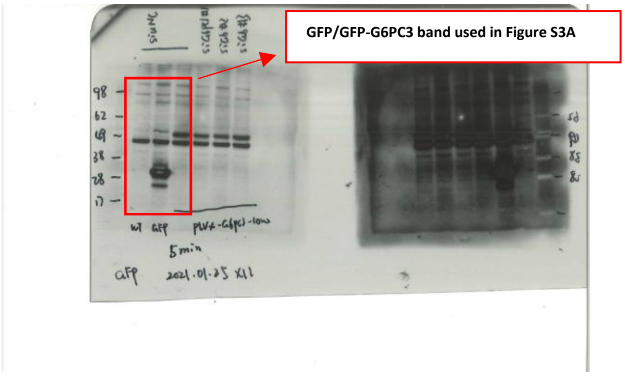

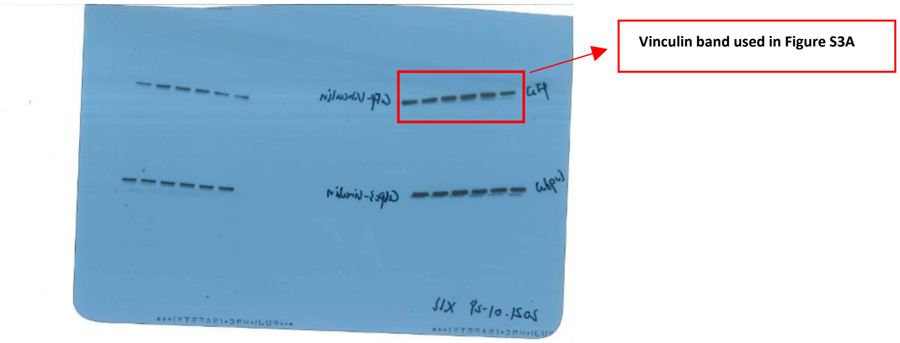

3. Full unedited gel for Figure S5B (from ChemiDoc Imager)
